# Biologic Monotherapies for Moderate-to-Severe Atopic Dermatitis: A Systematic Review and Bayesian Network Meta-Analysis of Established and Investigational Agents

**DOI:** 10.7759/cureus.105706

**Published:** 2026-03-23

**Authors:** Arya Babul, Devina Mehta, Yssra Soliman, Momina Hussain, Najib Babul

**Affiliations:** 1 Biomedical Sciences, West Career and Technical Academy, Las Vegas, USA; 2 Biomedical Sciences, Society for Awareness of Neglected Diseases, Las Vegas, USA; 3 General, Surgical, and Cosmetic Dermatology, Thomas Dermatology, Las Vegas, USA; 4 Genomics, Chinese Academy of Tropical Agriculture Sciences, Sanya, CHN; 5 Drug Development, Quadra Therapeutics, Las Vegas, USA

**Keywords:** atopic dermatitis, biological products, dupilumab, lebrikizumab, meta-analysis, rademikibart, rezpegaldesleukin, temtokibart, tralokinumab, zumilokibart

## Abstract

Biologic therapies targeting maladaptive type 2 inflammation have transformed the management of moderate‑to‑severe atopic dermatitis (AD); however, comparative evidence integrating both approved and next‑generation investigational agents remains limited. This Bayesian network meta‑analysis (BNMA) provides the first unified evaluation of all biologic monoclonal antibodies approved as monotherapy for AD (dupilumab, lebrikizumab, tralokinumab) together with emerging immunotherapies, including amlitelimab, rademikibart, rezpegaldesleukin, rocatinlimab, telazorlimab, temtokibart, and zumilokibart, across key efficacy measures.

A PRISMA‑2020-compliant systematic review and PROSPERO‑registered protocol (CRD420251162704) identified phase 2-3 randomized, double‑blind, placebo‑controlled trials reporting week‑16 outcomes (week‑24 for rocatinlimab). A Bayesian random‑effects NMA estimated relative risks (RRs) for EASI‑75, EASI‑90, IGA‑AD 0/1, and ≥4‑point improvement in itch Numeric Rating Scale (NRS). Treatment hierarchy was evaluated using both Bayesian (SUCRA) and frequentist (P‑score) approaches.

Seventeen randomized controlled trials (RCTs) involving more than 6,000 patients were included in the analysis. Dupilumab demonstrated the most consistent and reliable efficacy across all evaluated endpoints, including improvements in EASI-75, EASI-90, IGA-AD 0/1, and itch NRS. Among investigational therapies, rocatinlimab showed a notable signal for deep clinical responses, although the available evidence remains limited and less precise. Other emerging agents, including rademikibart, temtokibart, and zumilokibart, demonstrated encouraging efficacy profiles, whereas telazorlimab showed comparatively modest clinical benefit. Ranking analyses consistently positioned dupilumab as the most reliable and highest-performing therapy overall, followed by rocatinlimab. Overall, biologic therapies were generally well tolerated in the short term; however, dupilumab remains the only agent supported by extensive long-term safety data extending up to a decade.

This analysis relied on indirect comparisons anchored to short‑term placebo‑controlled induction periods, limiting assessment of long‑term safety and durability, particularly for mechanistically distinct agents such as rezpegaldesleukin, a regulatory T cell (Treg) pathway agonist, and rocatinlimab, an OXO pathway inhibitor. Evidence for several emerging therapies was restricted to small phase 2 trials, resulting in wide credible intervals (Crls) and lower certainty in comparative rankings. Trial heterogeneity in baseline severity, ethnic composition, and geographic setting may have introduced residual confounding despite sensitivity and meta‑regression analyses. These factors should be considered when interpreting relative efficacy estimates

This NMA demonstrates that dupilumab remains the most reliable and effective biologic monotherapy for moderate‑to‑severe AD, supported by the greatest precision, reproducibility, and long‑term safety. Rocatinlimab shows promising investigational efficacy but requires efficacy and long-term safety validation. Zumilokibart, rademikibart, and temtokibart emerge as additional candidates with encouraging activity, whereas telazorlimab showed limited clinical benefit. Collectively, these findings provide a comprehensive comparative framework to inform biologic selection and therapeutic sequencing.

## Introduction and background

Atopic dermatitis (AD) is a chronic inflammatory skin disease marked by pruritus, eczematous lesions, and sleep disturbance, affecting adolescents and adults worldwide [1, 2]. Persistent itch disrupts sleep, contributes to psychological distress, impairs quality of life, and imposes a substantial socioeconomic burden on patients and caregivers. Although topical therapies are effective for mild to moderate disease, many individuals with moderate-to-severe AD have inadequate control or recurrent flares and require systemic treatment [3]. Before the advent of dupilumab, which targets T helper 2 (Th2) pathways, management of refractory AD relied on high-potency topical corticosteroids, systemic corticosteroids, and conventional immunosuppressants, approaches supported by limited evidence and constrained by unfavorable long‑term safety profiles.

Type 2 immune dysregulation driven primarily by IL‑4, IL‑13, and IL‑31 is central to AD pathogenesis, contributing to skin‑barrier dysfunction, inflammation, and pruritus [4]. Dupilumab, a monoclonal antibody that blocks IL‑4 and IL‑13 signaling via IL‑4Rα, was the first biologic approved in the U.S. for moderate‑to‑severe AD across adults (2017), adolescents (2019), and children (2020) from six months of age [5-7]. Although highly effective, roughly two-thirds of patients do not achieve Eczema Area and Severity Index 90% improvement (EASI‑90) or Investigator’s Global Assessment for Atopic Dermatitis (0/1) (IGA 0/1) during the induction period, prompting the development of high‑affinity IL‑13 inhibitors. Tralokinumab (U.S. FDA approval 2021) and lebrikizumab (U.S. FDA approval 2024) both improve skin clearance, itch, and quality of life with favorable long-term safety profiles [8-15]. Zumilokibart (APG777), an investigational IL‑13 inhibitor, demonstrates affinity comparable to lebrikizumab, early efficacy, and the potential for markedly reduced injection frequency. Nemolizumab, an IL‑31Rα antagonist, is the most recently introduced biologic (U.S. FDA approval 2024) for adults and adolescents with moderate‑to‑severe AD [16], though Phase 3 trials evaluated it only in combination with topical corticosteroids and/or calcineurin inhibitors. Overall, biologics are preferred to conventional immunosuppressants due to their targeted mechanisms, minimal systemic toxicity, and limited laboratory monitoring requirements. Differences in biologic response among patients with AD likely reflect underlying disease heterogeneity, including variation in immune pathways, skin-barrier integrity, microbiome profiles, genetic factors, and environmental exposures. These differences may help explain the modest EASI-90 response rates (around 30%-40%) reported in clinical trials [4, 17]. Although Th2‑driven inflammation predominates, subsets of patients exhibit Th1, Th17, or Th22 skewing [4, 18]. These observations have accelerated interest in investigational immunotherapies such as rocatinlimab and telazorlimab, anti‑OX40 antibodies that inhibit pathogenic T‑cell activity, and amlitelimab, an anti‑OX40L antibody that disrupts costimulatory signaling on antigen‑presenting cells [18-23].

Rezpegaldesleukin (NKTR-358), a first‑in‑class IL‑2 pathway agonist, expands regulatory T cells (Treg) and modulates inflammatory cytokine activity, with recent evidence of efficacy in severe alopecia areata and moderate‑to‑severe AD [24-26]. Alongside dupilumab, it is one of the few agents reported to improve both AD and comorbid asthma in clinical studies [25]. Its therapeutic rationale is grounded in the coordinated immune‑resolving functions of Tregs, a biology underscored by the 2025 Nobel Prize in Physiology or Medicine recognizing the discovery of Tregs [27-30]. This mechanism differs from cytokine‑blocking biologics, which inhibit individual inflammatory pathways. However, the extent to which rezpegaldesleukin’s Treg-modulating activity directly accounts for its clinical effects in AD remains to be determined.

Zumilokibart is a humanized IgG1 mAb with high IL‑13 affinity and an extended half‑life [31, 32]. Rademikibart, another investigational agent targeting IL‑4Rα, blocks IL‑4 and IL‑13 signaling and is currently under review by China’s National Medical Products Administration (NMPA) for moderate‑to‑severe AD in adults and adolescents [4, 33-35]. Temtokibart blocks IL-22R1 within the Th22 pathway and has shown efficacy in AD [36]. Collectively, these emerging therapies introduce novel mechanisms and have the potential to offer more durable or deeper immunologic remission.

Biologic therapies for AD have only recently entered clinical practice, and direct non‑inferiority trials are scarce and unlikely to be undertaken given the logistical and financial demands of detecting modest differences among already effective agents. In this setting, indirect statistical approaches are essential for establishing comparative efficacy. Network meta‑analysis (NMA) enables cross‑trial comparisons by anchoring treatments to a shared comparator, typically a placebo, a design well-suited to AD, where placebo‑controlled trials are common, and supports provisional treatment rankings for both efficacy and safety.

Previous NMAs in AD, including the comprehensive NMA by Chu et al. [37], the living analyses by Drucker et al. [38, 39], and the series by Silverberg et al. [40, 41] have compared approved biologics and oral JAK inhibitors but have not incorporated the newest investigational immunotherapies [13, 37-41]. Earlier work focused largely on established biological and small‑molecule agents. Although some emerging therapies have been discussed in selected reviews, others, such as rezpegaldesleukin, zumilokibart, and temtokibart, have not been systematically evaluated. To our knowledge, no prior meta‑analysis has integrated all of these agents within a unified monotherapy network. Our NMA expands the evidence base by jointly analyzing approved biologics (dupilumab, tralokinumab, and lebrikizumab) alongside next‑generation investigational immunotherapies (amlitelimab, rademikibart, rezpegaldesleukin, rocatinlimab, telazorlimab, temtokibart, and zumilokibart), providing the first comprehensive comparative ranking of advanced monotherapies for adolescents and adults with moderate‑to‑severe AD.

This study informs clinical decision‑making, clarifies the relative positioning of emerging immunotherapies, and identifies evidence gaps that warrant future head‑to‑head trials.

## Review

Methods

Study Selection and Data Sources

A systematic review conducted in accordance with Preferred Reporting Items for Systematic Reviews and Meta-Analyses (PRISMA) 2020 and registered in International Prospective Register of Systematic Reviews (PROSPERO; registration number: CRD420251162704) [42] included phase 2 and 3 randomized, double-blind, placebo-controlled trials of biologics for moderate-to-severe AD. Searches of PubMed, Cochrane, ScienceDirect, and ClinicalTrials databases through September 2025, plus key references and congress abstracts, identified eligible studies.

Eligibility Criteria

Eligible treatments included lebrikizumab (250 mg), rocatinlimab (300 or 600 mg), dupilumab (300 mg), rezpegaldesleukin (24 µg/kg or 18 µg/kg), amlitelimab (250 mg), tralokinumab (300 mg), telazorlimab (75, 300, or 600 mg), temtokibart (450 mg), rademikibart (300 mg), and zumilokibart (360 mg). Age criteria aligned with pivotal programs: lebrikizumab (Advocate 1 (NCT04146363); Advocate 2 (NCT04178967); Phase 2b Study (NCT03443024) ≥12 years), rocatinlimab (OX40 (NCT03703102); ROCKET-Horizon (NCT05651711) ≥18 years), dupilumab (SOLO-1 (NCT02277743); SOLO-2 (NCT02277769); phase 2b study (NCT01859988) ≥18 years), rezpegaldesleukin REZOLVE-AD (NCT06136741) >18, amlitelimab (phase 2b study (NCT05131477) ≥18 years), tralokinumab (phase 2b study (NCT02347176); ECZTRA 1 (NCT03131648); ECZTRA 2 (NCT03160885); ECZTRA 6 (NCT03526861) 12-17, 18-75 years), telazorlimab (Phase 2b Study (NCT03568162) ≥18 years), temtokibart (Phase 2a study (NCT04922021) 18-64 years), rademikibart (CBP-201 (NCT05017480) 12-75 years), and zumilokibart (APEX Phase 2 Part A (NCT06395948) ≥18 years) [5, 6, 8-12, 19-22, 32, 34, 36, 43]. These seven biologics were chosen for their clinical and regulatory importance as biologic monotherapies for moderate-to-severe AD. Approved biologics and investigational agents were administered according to their respective pivotal trial regimens; for consistency, maintenance doses are referenced throughout the manuscript text, while loading and maintenance schedules are detailed in the tables.

Efficacy Outcomes

Efficacy outcomes of interest were consistent across all randomized controlled trials (RCTs). Included studies either employed the validated IGA-AD (vIGA-AD) scale [44-46] or did not specify the source of their IGA scale [47-50]. The primary endpoints were (i) the (i) proportion of patients achieving at least EASI-90; (ii) EASI 75% improvement (EASI-75) [51]; (iii) the proportion achieving a score of 0 (clear) or 1 (almost clear) on the IGA-AD 0/1, with a minimum two-point improvement from baseline [52]; and (iv) the proportion of patients with a ≥4-point reduction in the itch Numeric Rating Scale (NRS) score [53]. Baseline disease severity eligibility criteria for each trial arm are summarized in Table 1. These criteria were applied at baseline only to determine participant eligibility. Clinical efficacy endpoints were then reassessed at the primary endpoint evaluation timepoint, which occurred at week 16 for lebrikizumab, dupilumab, rezpegaldesleukin, amlitelimab, tralokinumab, zumilokibart, telazorlimab, temtokibart, and rademikibart, and at week 24 for rocatinlimab. Not all trials reported all efficacy endpoints; in particular, EASI-90 and/or Itch NRS outcomes were unavailable for some investigational agents. Consequently, analyses for these outcomes were conducted using all available data, and treatment comparisons for EASI-90 and itch improvement reflect a reduced evidence base for selected agents.

**Table 1:** Included studies and treatment doses evaluated in the network meta-analysis Abbreviations: APG777, Zumilokibart; NCT, National Clinical Trial unique identifier assigned to studies registered on ClinicalTrials.gov; RCT, randomized controlled trial; q2w/q4w, every two/four weeks; US, United States; UK, United Kingdom; mg, milligrams; µg/kg, micrograms per kilogram *Approved biologics include an initial loading/induction phase followed by maintenance dosing. For consistency, full dosing regimens (loading and maintenance) are presented in tables, while the manuscript text refers to maintenance doses.

Study ID	Trial name	Study Design	Setting	Endpoint	Intervention arm	Total Sample (n)
Silverberg, 2023 [12]	Advocate 1 (NCT04146363)	Identical, randomized, double-blinded, placebo-controlled, parallel-group, monotherapy phase III trials	89 centers in the US, Bulgaria, Canada, Germany, Mexico, Singapore, Taiwan, Ukraine	16	Lebrikizumab: 500 mg at Weeks 0 and 2, followed by 250 mg (q2w) ^*^	283
					Placebo	141
	Advocate 2 (NCT04178967)				Lebrikizumab: 500 mg at Weeks 0 and 2, followed by 250 mg (q2w)^*^	281
					Placebo	146
Guttman-Yassky, 2020 [11]	Phase 2b Study (NCT03443024)	A phase 2b, double-blind, placebo-controlled, dose-ranging randomized clinical trial	57 US centers	16	Lebrikizumab: 500 mg at Weeks 0 and 2, followed by 250 mg (q2w)^*^	75
					Placebo	52
Simpson, 2016 [6]	SOLO-1 (NCT02277743)	Independent, randomized, double-blind, placebo-controlled, parallel-group trials	North America, Europe, Asia	16	Dupilumab: 600 mg loading dose at Week 0, followed by 300 mg (q2w)^*^	223
					Placebo	224
	SOLO-2 (NCT02277769)				Dupilumab: 600 mg loading dose at Week 0, followed by 300 mg (q2w)^*^	239
					Placebo	236
Thaci, 2016 [5]	Phase 2b study (NCT01859988)	Randomized, placebo-controlled, double-blind dose-ranging phase 2b study	91 centers in Canada, Czechia, Germany, Hungary, Japan, Poland, the US	16	Dupilumab: 600 mg loading dose at Week 0, followed by 300 mg (q2w)^*^	64
					Placebo	61
Wollenberg, 2021 [9]	ECZTRA 1 (NCT03131648)	Randomized, double-blind, placebo-controlled, phase III trials	123 centers in the US, Europe, and Asia	16	Tralokinumab: 600 mg loading dose, followed by 300 mg (q2w)^*^	603
					Placebo	199
	ECZTRA 2 (NCT03160885)		115 centers in the US, Australia, Canada, the UK, Europe, and Asia	16	Tralokinumab: 600 mg loading dose, followed by 300 mg (q2w)^*^	593
					Placebo	201
Peller, 2023 [8]	ECZTRA 6 (NCT03526861)	Randomized, double-blinded, placebo-controlled	72 centers in North America, Europe, Asia, Australia	16	Tralokinumab: 600 mg loading dose, followed by 300 mg (q2w)^*^	97
					Placebo	94
Weidinger, 2025 [21]	Phase 2b study (NCT05131477)	Two-part, phase 2b, randomized, double-blinded, placebo-controlled trial	100 centers in the US, Australia, Bulgaria, Canada, Czechia, Germany, Hungary, Japan, Poland, Spain, Taiwan, UK	16	Amlitelimab 250 mg (q4w) (plus loading dose)	77
					Placebo	79
Guttman-Yassky, 2023, 2025 & Abdelhalim, 2024 [19, 20, 23]	OX40/Horizon (NCT03703102)	Phase 2b, multicenter, randomized, double-blind, parallel-group study	58 centers in the US, Canada, Japan, and Germany	16	Rocatinlimab 150 mg (q4w) (dose per trial arm)	54
					Rocatinlimab 600 mg (q4w) (dose per trial arm)	54
					Rocatinlimab 300 mg (q2w) (dose per trial arm)	55
					Rocatinlimab 600 mg (q2w) (dose per trial arm)	54
					Placebo	57
Guttman-Yassky, 2025 [20]	IGNITE (NCT05398445)	Phase 3, randomized, double-blind study	202 centers in Argentina, Brazil, Canada, China, Croatia, Czechia, Germany, Greece, Hungary, Italy, Japan, Latvia, Netherlands, Poland, Portugal, Puerto Rico, Slovakia, South Korea, Spain, Taiwan	24	Rocatinlimab 150 mg (q4w) (dose per trial arm)	760
					Rocatinlimab 300 mg (q4w) (dose per trial arm)	
					Placebo	
Silverberg, 2025 [43]	REZOLVE-AD (NCT06136741)	Randomized, Phase 2b, double-blind	107 centers in the US, Australia, Bulgaria, Canada, Croatia, Czechia, Germany, Hungary, Poland, and Spain	16	Rezpegaldesleukin 24 µg/kg (q2w)	104
					Rezpegaldesleukin 18 µg/kg (q2w)	106
					Rezpegaldesleukin 24 µg/kg (q4W)	110
					Placebo	73
Henderson, 2025 [32]	APEX Phase 2 Part A (NCT06395948)	Two-part, multicenter, randomized, placebo-controlled phase 2 study	93 centers in the US, Czechia, France, Germany, Hungary, Poland, Spain, Canada, UK	16	APG777 720 mg at Weeks 0 and 2, followed by 360 mg at Weeks 4 and 12	82
					Placebo	41
Zhang, 2025 [34]	CBP-201 (NCT05017480)	SEASIDE CHINA phase II, randomized, double-blind, placebo-controlled trial	48 centers in China	16	Rademikibart 300 mg (q2w)	219
					Placebo	111
Thaci, 2025 [36]	Phase 2a study (NCT04922021)	Phase 2a, randomized, double-blind, placebo-controlled	17 centers in the US, Canada, Germany, and Poland	16	Temtokibart 450 mg (q2w) (dose per trial arm)	29
					Placebo	29
Rewerska, 2023 [22]	Phase 2b Study (NCT03568162)	Randomized, double-blind, placebo-controlled, parallel-group study	95 centers in 83 centers in the US, Canada, Czechia, Germany, and Poland	16	Telazorlimab 300 mg (q2w)	76
					Telazorlimab 300 mg (q4w)	78
					Telazorlimab 75 mg (q4w)	77
					Placebo	80
					Telazorlimab 600 mg (q2w)	75
					Placebo	74

Statistical Analysis

A Bayesian NMA (BNMA) compared the relative efficacy of 10 biologic monotherapies versus placebo. Analyses were performed using OpenBUGS v3.2.3 (MRC Biostatistics Unit at the University of Cambridge, United Kingdom) within R v4.2.2 (R Core Team at the R Foundation for Statistical Computing, Vienna, Austria) using the R2OpenBUGS package (maintained by Martyn Plummer) to interface R with OpenBUGS [54-56]. Fixed- and random-effect models, with and without baseline risk adjustment, were compared using the deviance information criterion (DIC) [57]. Relative risk (RR) with 95% credible intervals (CrIs) was estimated, and treatment hierarchy was assessed using P-scores and Surface Under the Cumulative Ranking Curve (SUCRA) values [17, 58-62]. Risk of bias was evaluated using the Cochrane Risk of Bias tool, version 2.0 (RoB 2.0) [63-65], following established methodological and BNMA reporting guidelines [11, 66]. In accordance with the updated Risk Of Bias in Network Meta-Analysis 2025 (ROB-NMA 2025) guidelines [66], review-level bias was assessed using the Risk Of Bias In Systematic reviews (ROBIS) tool, and network-specific bias was evaluated using the ROB-NMA framework. To ensure adequate statistical power and enable consistent cross-drug comparison, efficacy data were pooled across all available phase 2 and phase 3 trials for each biologic using published aggregate data only. For each drug, identical dosing regimens, timepoints (week 16), and outcome definitions (EASI-75, EASI-90, Investigator’s Global Assessment for Atopic Dermatitis 0/1 (IGA‑AD 0/1), and itch NRS ≥4-point response) were combined by aggregating the reported numbers of responders and total participants across studies. Placebo groups were pooled using the same approach to generate a unified comparator arm for each drug class. This method allowed standardized calculation of pooled response rates and improved precision of effect estimates, particularly for agents evaluated in multiple trials with varying sample sizes. No individual patient-level data were accessed or analyzed at any stage.

Ethical Approval

Ethical approval and patient consent were not required because this was an NMA of previously published studies.

Results

Systematic Literature Review

**Figure 1:**
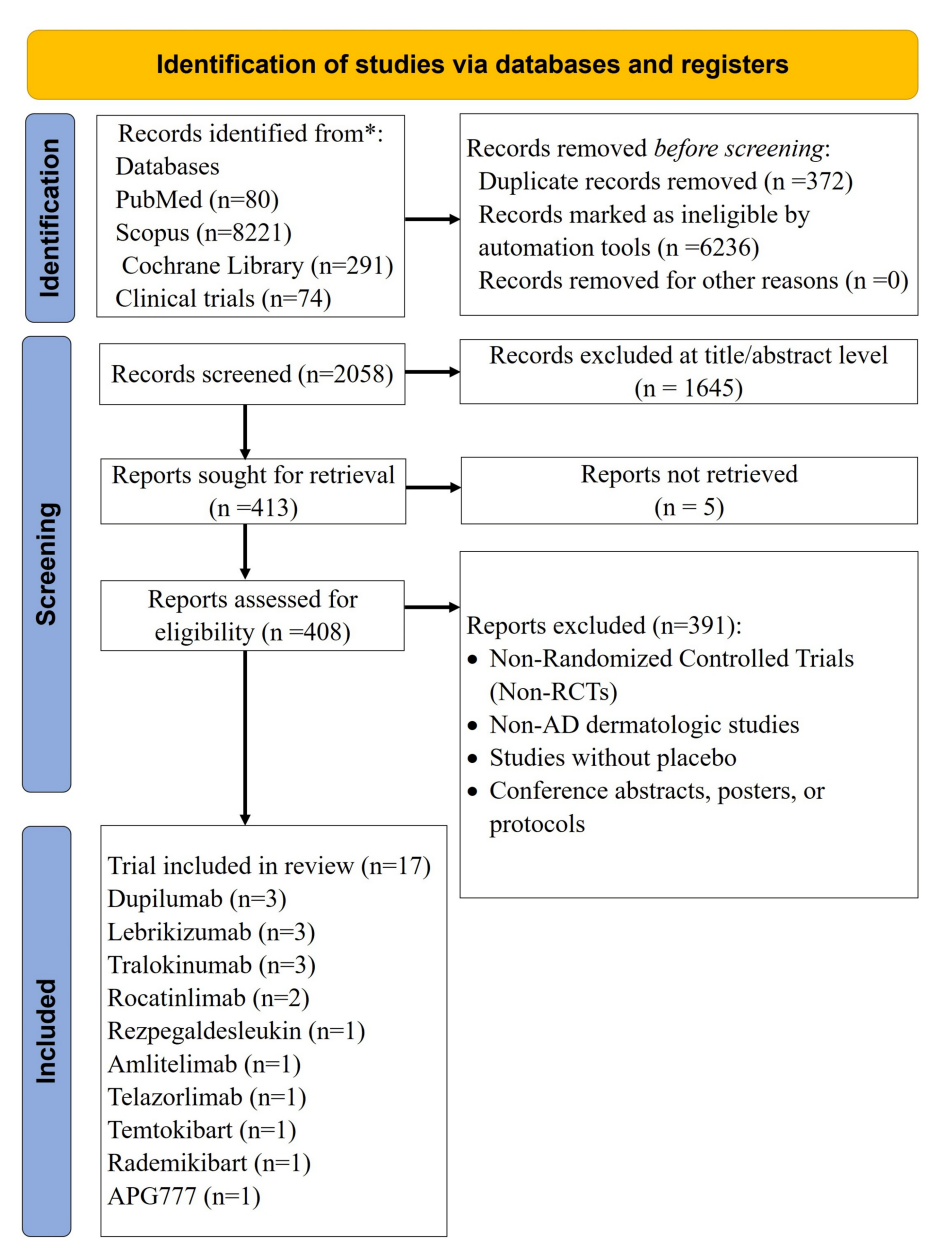
A PRISMA Flowchart Outlining the Study Selection Process The flow diagram summarizes identification, screening, eligibility assessment, and inclusion of randomized trials evaluating biologic and investigational therapies for atopic dermatitis. Abbreviation: APG777, zumilokibart; PRISMA: Preferred Reporting Items for Systematic Reviews and Meta-Analyses

By means of a systematic literature search, 8,666 records were found. Of these, 2,058 records were screened for their eligibility, and 413 records had unique findings. In total, 17 RCTs from 14 publications were eventually included in the NMA (Figure 1). For moderate-to-severe AD, these 17 studies included 6,366 patients who were given either a placebo or one of the following biologic monotherapies: lebrikizumab (250 mg), rocatinlimab (300/600 mg), dupilumab (300 mg), rezpegaldesleukin (24 ug/kg/18 ug/kg), amlitelimab (250 mg), tralokinumab (300 mg), telazorlimab (75/300/600 600 mg), temtokibart (450 mg), rademikibart (300 mg), and zumilokibart (360 mg) [5, 6, 8-12, 19-21, 32-34, 36, 43]. All 17 studies were double-blind, randomized, placebo-controlled phase 2 or phase 3 clinical trials, published between 2015 and 2025. One study (rocatinlimab 300 mg every four weeks (q4w)) had a 24-week placebo-controlled duration [20]; the remaining 16 studies had a 16-week placebo-controlled duration (Table 1). The pooled population had balanced sex distribution (28.8%-65.3% female), mean age 14.0-42.2 years, and racially diverse representation. Baseline EASI scores (25.2%-33.8), IGA-AD scores (29.1-51.3), and severe Itch NRS score (6.34-8.1) reflected moderate-to-severe disease, with a mean disease duration of 13.0-30.5 years (Table 2).

**Table 2:** Characteristics of the included trials and treatment regimens Abbreviations: A, Asian; AI/AN, American Indian/Alaska Native; APG777, Zumilokibart; B, Black; C, Chinese; EASI, Eczema Area and Severity Index; IGA-AD, Investigator’s Global Assessment for Atopic Dermatitis; J, Japanese; M, Mixed; NA, not available; NCT, National Clinical Trial unique identifier assigned to studies registered on ClinicalTrials.gov; NRS, Numerical Rating Scale; O, Other; q2w/q4w, every two/four weeks; SD, standard deviation; W, White *Approved biologics include an initial loading/induction phase followed by maintenance dosing. For consistency, full dosing regimens (loading and maintenance) are presented in tables, while the manuscript text refers to maintenance doses.

Trial name	Drugs/Dose	Total Patients (n)	Age, mean (±SD), years	Female, n (%)	Race (%)	Disease onset, mean (SD), years	EASI score mean (SD)	Severe IGA-AD score, mean (SD)	Severe Itch NRS, mean (SD)
Advocate 1 (NCT04146363) [12]	Lebrikizumab: 500 mg at Weeks 0 and 2, followed by 250 mg (q2w)^*^	283	36.1 (17.8)	141 (49.8)	A: 13.8, B: 11.7, O: 5.3, W: 69.3	22.0 (14.9)	28.8 (11.3)	113 (39.9)	7.2 (1.9)
	Placebo	141	34.2 (16.4)	73 (51.8)	A: 22, B: 11.3, O: 0.7, W: 66	23.8 (15.4)	31.0 (12.9)	58 (41.1)	7.3 (1.7)
Advocate 2 (NCT04178967) [12]	Lebrikizumab: 500 mg at Weeks 0 and 2, followed by 250 mg (q2w)^*^	281	36.6 (16.8)	136 (48.4)	A: 27.8, B: 8.9, O: 3.6, W: 59.8	20.8 (15.2)	29.7 (12.0)	106 (37.7)	7.1 (1.9)
	Placebo	146	35.3 (17.2)	75 (51.4)	A: 30.1, B: 6.8, O: 4.8, W: 58.2	20.1 (14.1)	29.6 (10.8)	51 (34.9)	7.2 (1.9)
Phase 2b Study (NCT03443024) [11]	Lebrikizumab: 500 mg at Weeks 0 and 2, followed by 250 mg (q2w)^*^	75	38.9 (17.4)	49 (65.3)	A: 8.0, AI/AN: 1.3, B: 30.7, M/O: 6.7, W: 53.3	22.1 (17.2)	25.5 (11.2)	22 (29.3)	7.6 (1.9)
	Placebo	52	42.2 (18.2)	24 (46.2)	A: 11.5, AI/AN: 0, B: 30.8, M/O: 7.7, W: 50.0	24.4 (17.4)	28.9 (11.8)	20 (38.5)	7.4 (2.4)
SOLO-1 (NCT02277743) [6]	Dupilumab: 600 mg loading dose at Week 0, followed by 300 mg (q2w)^*^	223	38	94 (42.2)	A: 24, B: 4, O: 2, W: 69	26	30.4	108 (48)	7.6
	Placebo	224	39	105 (46.6)	A: 25, B: 7, O: 3, W: 65	28	31.8	110 (49)	7.7
SOLO-2 (NCT02277769) [6]	Dupilumab: 600 mg loading dose at Week 0, followed by 300 mg (q2w)^*^	239	40	96 (40.2)	A: 19, B: 6, O: 5, W: 71	24.5	28.6	115 (49)	7.8
	Placebo	236	35	104 (44.1)	A: 21, B: 8, O: 4, W: 66	26	30.5	115 (49)	7.7
Phase 2b study (NCT01859988) 6 [5]	Dupilumab: 600 mg loading dose at Week 0, followed by 300 mg (q2w)^*^	64	39.4 (12.1)	23 (35.9)	NA	30.5 (13.8)	33.8 (14.5)	30 (47)	6.74 (1.57)
	Placebo	61	37.2 (13.1)	21 (34.4)	NA	29.8 (13.5)	32.9 (13.8)	29 (48)	6.34 (1.83)
ECZTRA 1 (NCT03131648) [9]	Tralokinumab: 600 mg loading dose, followed by 300 mg (q2w)^*^	603	37	252 (41.8)	A: 19.9, B: 6.8, O: 2.6, W: 70.6	27	28.1	301 (50.0)	7.9
	Placebo	199	37	76 (38.2)	A: 20.1, B: 9.0, O: 1.5, W: 69.3,	28	30.3	102 (51.3)	7.9
ECZTRA 2 (NCT03160885) [9]	Tralokinumab: 600 mg loading dose, followed by 300 mg (q2w)^*^	593	34	234 (39.5)	A: 26.0, B: 7.3, O: 3.7, W: 63.1	25	28.2	286 (48.2)	8
	Placebo	201	30	87 (43.3)	A: 25.9, B: 8.5, O: 4.5, W: 61.2,	25	29.6	101 (50.2)	8.1
ECZTRA 6 (NCT03526861) [8]	Tralokinumab: 600 mg loading dose, followed by 300 mg (q2w)^*^	97	15	50 (51.5)	NA	13	28	48 (49.5)	8.1
	Placebo	94	14	43 (45.7)	NA	13	27.2	43 (45.7)	7.6
Phase 2b study (NCT05131477) [21]	Amlitelimab 250 mg (q4w)	77	36.3 (13.3)	30 (39.0)	A: 15.6, B: 5.2, O: 0, W: 79.2	22.0 (15.4)	30.3 (11.7)	24 (31.2)	7.3 (1.30)
	Placebo	79	36.4 (13.1	30 (38.0)	W: 75.9, B: 7.6, A: 15.2, O: 1.3	22.2 (16.6)	26.4 (7.9)	23 (29.1)	7.4 (1.39)
OX40 (NCT03703102) [19, 20, 23]	Rocatinlimab 150 mg (q4w)	54	37.2 (13.8)	16 (28.8)	A: 11.5, B: 5.8, J: 55.8, O: 1.9 W: 25.0	18·9 (16·2)	33·2 (13·1)	23 (44)	7·7 (1·6)
	Rocatinlimab 600 mg (q4w)	54	39.1 (14.6)	23 (42.3)	A: 7.7, B: 1.9, J: 51.9, O: 0, W: 38.5	16·1 (15·5)	32·5 (12·8)	24 (46)	7·7 (2·1)
	Rocatinlimab 300 mg (q2w)	55	37.2 (14.4)	23 (42.3)	J: 59.6, A: 7.7, B: 3.8, W: 28.8, O: 0	15·0 (12·5)	31·6 (12·5)	22 (42)	7·3 (1·6)
	Rocatinlimab 600 mg (q2w)	54	37.3 (16.3)	24 (44.4)	A: 5.6, B: 1.9, J: 55.6, O: 0, W: 37.0	15·2 (13·1)	31·1 (11·8)	25 (46)	7·6 (1·9)
	Placebo	57	38.7 (14.4)	26 (45.6)	J: 52.6, A: 12.3, B: 10.5, W: 24.6, O: 0	15.8 (17.0)	29.2 (13.3)	26 (46)	7·2 (2·3)
IGNITE (NCT05398445) [20]	Rocatinlimab 150/300 mg (q4w)	760	NA	NA	NA	NA	NA	NA	NA
REZOLVE-AD (NCT06136741) [43]	Rezpegaldesleukin 24 µg/kg (q2w)	104	38.0 (13.73)	49 (47.1)	W: 26 O: 74	NA	25.4 (9.14)	33 (31.7)	6.8 (2.0)
	Rezpegaldesleukin 18µg/kg (q2w)	106	36.3 (15.41)	56 (52.8)	W: 27.4, 72.6	NA	27.2 (10.40)	36 (34.0)	6.7 (1.9)
	Rezpegaldesleukin 24 µg/kg (q4W)	110	36.5 (14.30)	63 (57.3)	W: 31.84, O: 68.16	NA	26.1 (10.45)	35 (31.8)	7.1 (1.8)
	Placebo	73	37.9 (14.39)	35 (47.9)	W: 28.8, O: 71.2	NA	25.2 (8.57)	22 (30.1)	6.3 (2.2)
APEX Phase 2 Part A (NCT06395948) [32]	APG777 720 mg at Weeks 0 and 2, followed by 360 mg at Weeks 4 and 12	82	38.7 (15.6)	41 (50.0)	A: 14.6, B: 15.9, O: 3.7, W: 65.9	24.2 (14.5)	25.2 (10.8)	27 (32.9)	6.4 (2.1)
	Placebo	41	36.0 (13.7)	19 (46.3)	A: 9.8, B: 14.6, O: 2.4, W: 73.2	24.6 (14.1)	25.3 (10.8)	14 (34.1)	6.7 (1.9)
CBP-201 (NCT05017480) [34]	Rademikibart 300 mg (q2w)	219	38.6 (16.65)	73 (33.3)	C: (100)	10.1 (7.85)	29.27 (11.66)	120 (54.8)	7.19 (1.68)
	Placebo	111	39.6 (17.54)	40 (36)	C: (100)	11.3 (8.12)	28.62 (12.09)	61 (55.0)	7.17 (1.46)
Phase 2a study (NCT04922021) [36]	Temtokibart 450 mg (q2w) (dose per trial arm)	29	38.1 (12.1)	17 (58.6)	A: 13.8, B: 0, W: 84.5	26.9 (13.0)	26.9 (11.8)	12 (41.4)	7.6 (1.6)
	Placebo	29	32.8 (10.2)	16 (55.2)	A: 6.9, B: 10.3, W: 82.8	23.4 (12.3)	26.1 (9.8)	9 (31.0)	7.5 (1.6)
Phase 2b Study (NCT03568162) [22]	Telazorlimab 300 mg (q2w)	76	40.2 (13.1)	32 (42.1)	A: 6.6, B: 17.1, M: 0, O: 0, W: 76.3	27.7 (15.8)	30.4 (14.1)	27 (35.5)	7.4 (1.6)
	Telazorlimab 300 mg (q2w)	78	36.6 (14.8)	44 (56.4)	A: 5.1, B: 17.9, M: 1.3, O: 0, W: 75.6	29.7 (15.6)	33.8 (14.9)	30 (38.5)	7.5 (1.7)
	Telazorlimab 75 mg (q2w)	77	38.4 (16.9)	41 (53.2)	A: 5.2, B: 7.8, M: 2.6, O: 0, W: 84.4	25.3 (14.1)	28.4 (11.6)	28 (36.4)	7.5 (1.6)
	Placebo	80	36.3 (13.1)	44 (55.0)	A: 5.2, B: 7.8, M: 2.6, O: 0, W: 84.4	28.4 (14.4)	30.7 (13.2)	28 (35.0)	7.3 (1.8)
	Telazorlimab 600 mg (q2w)	75	37.9 (13.3)	37 (49.3)	A: 2.7, B: 8.0, M: 0, O: 1.3, W: 88.0	27.6 (16.5)	29.9 (13.2)	27 (36.0)	7.4 (1.5)
	Placebo	74	36.0 (13.8)	47 (63.5)	A: 1.4, B: 5.4, M: 0, O: 0, W: 93.2	27.9 (15.4)	31.8 (14.3)	27 (36.5)	7.4 (1.7)

Feasibility Assessment, Network Fit, and Risk of Bias

**Figure 2:**
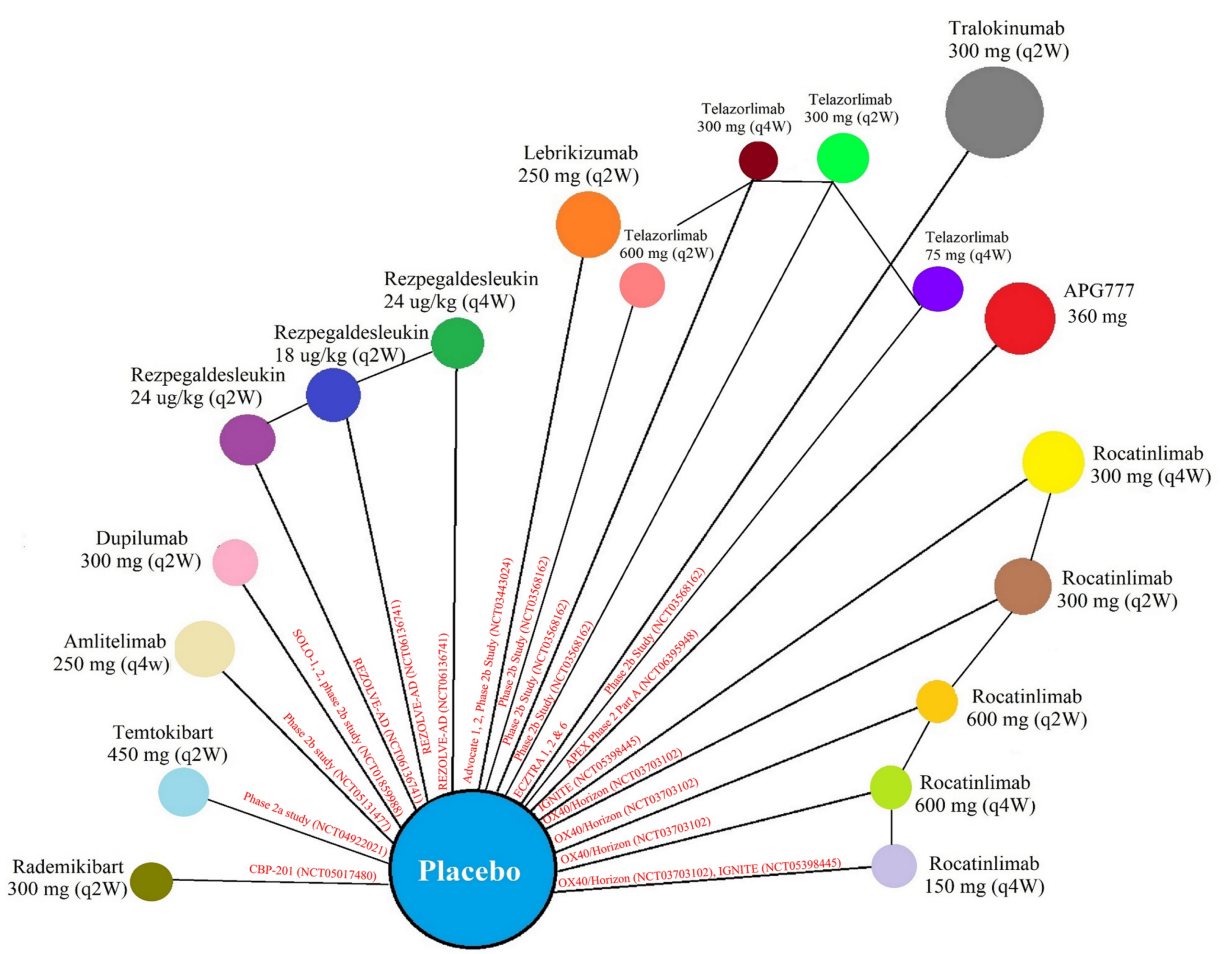
Network Geometry of the Included Trials Network plot showing treatment nodes proportional to sample size and direct comparisons linking each regimen to placebo across all included randomized trials. Treatment nodes represent maintenance dosing regimens. Approved biologics were administered with loading doses (LD) followed by maintenance therapy: dupilumab (600 mg LD, then 300 mg every two weeks (q2w)), lebrikizumab (500 mg LD at Weeks 0 and 2, then 250 mg q2w), and tralokinumab (600 mg LD, then 300 mg q2w). Investigational agents were administered according to trial-specific induction and maintenance schedules. Abbreviations: APG777, Zumilokibart. Advocate 1 (NCT04146363) [12]; Advocate 2 (NCT04178967) [12]; Phase 2b Study (NCT03443024) [11]; SOLO-1 (NCT02277743) [6]; SOLO-2 (NCT02277769) [6]; Phase 2b Study (NCT01859988) [5]; ECZTRA 1 (NCT03131648) [9]; ECZTRA 2 (NCT03160885) [9]; ECZTRA 6 (NCT03526861) [8]; Phase 2b Study (NCT05131477) [21]; OX40 (NCT03703102) [19, 20, 23]; IGNITE (NCT05398445) [20]; REZOLVE-AD (NCT06136741) [43]; APEX Phase 2 Part A (NCT06395948) [32]; CBP-201 (NCT05017480) [34]; Phase 2a Study (NCT04922021) [36]; Phase 2b Study (NCT03568162) [22] Image credits: The network plot was generated by the authors using R (version 4.4.2; R Core Team at the R Foundation for Statistical Computing, Vienna, Austria) with the netmeta package. Visualization and graphical customization were performed in RStudio (version 2024.12.0;  Posit, PBC, Boston, MA, USA) using the netgraph function, with color-coding, node sizing, and edge thickness applied to enhance interpretability of the treatment network.

**Figure 3:**
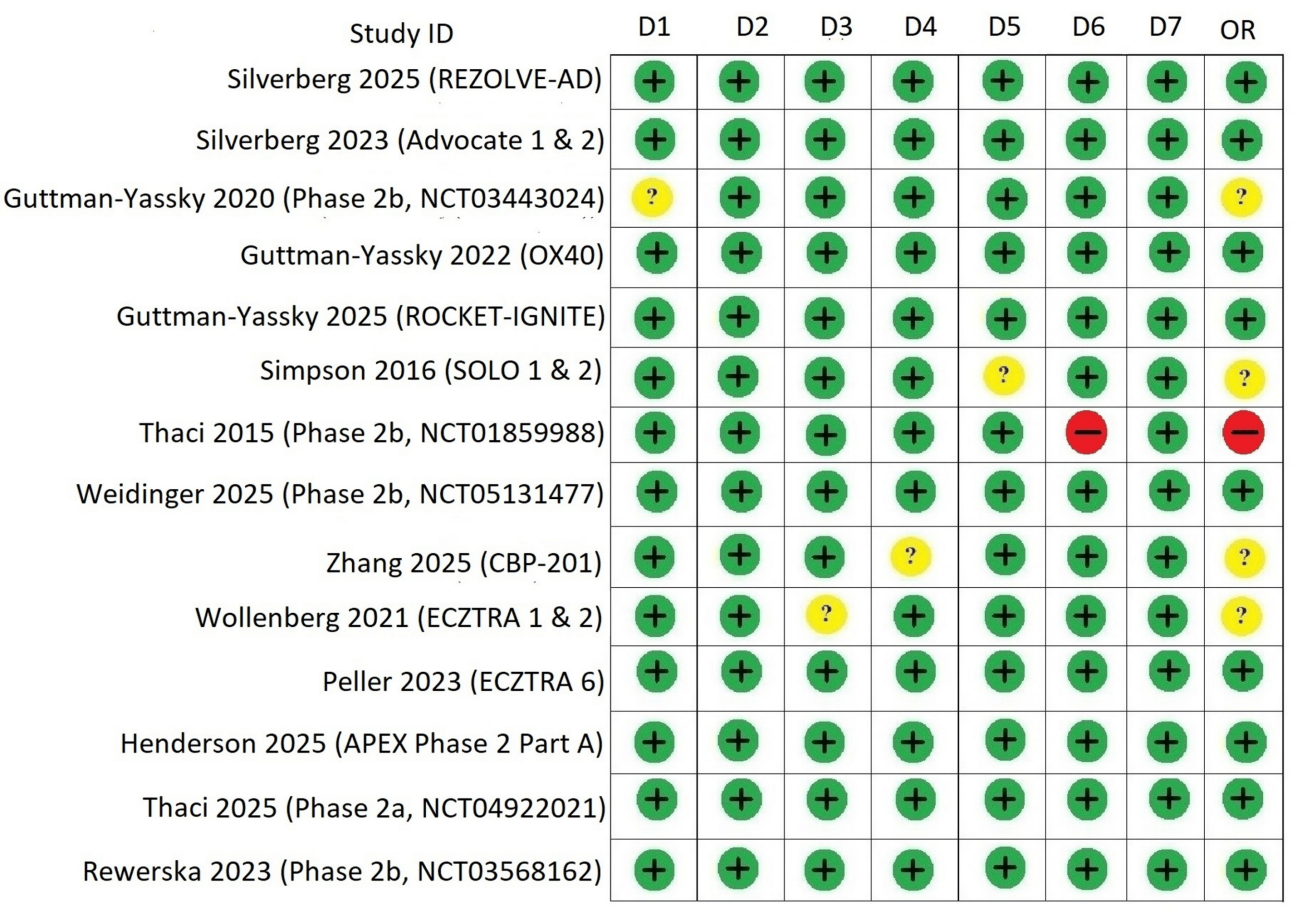
Risk of bias assessment of included randomized controlled trials (seven-domain Cochrane tool) Risk of bias was assessed across seven domains: D1, random sequence generation (selection bias); D2, allocation concealment (selection bias); D3, blinding of participants and personnel (performance bias); D4, blinding of outcome assessment (detection bias); D5, incomplete outcome data (attrition bias); D6, selective reporting (reporting bias); and D7, other sources of bias. Each domain was judged as low, unclear, or high risk of bias, with an overall risk-of-bias (OR) assessment derived for each study. Trials evaluated biologic therapies administered with protocol-specified loading doses (LDs) followed by maintenance dosing (dupilumab: 600 mg LD → 300 mg every two weeks (q2w); lebrikizumab: 500 mg LD at Weeks 0 and 2 → 250 mg q2w; tralokinumab: 600 mg LD → 300 mg q2w) and investigational agents administered according to trial-specific induction and maintenance regimens. Abbreviations: APG777, Zumilokibart; CBP-201, Rademikibart; NCT, National Clinical Trial unique identifier assigned to studies registered on ClinicalTrials.gov. Advocate 1 (NCT04146363) [12]; Advocate 2 (NCT04178967) [12]; Phase 2b Study (NCT03443024) [11]; SOLO-1 (NCT02277743) [6]; SOLO-2 (NCT02277769) [6]; Phase 2b Study (NCT01859988) [5]; ECZTRA 1 (NCT03131648) [9]; ECZTRA 2 (NCT03160885) [9]; ECZTRA 6 (NCT03526861) [8]; Phase 2b Study (NCT05131477) [21]; OX40 (NCT03703102) [19, 20, 23]; IGNITE (NCT05398445) [20]; REZOLVE-AD (NCT06136741) [43]; APEX Phase 2 Part A (NCT06395948) [32]; CBP-201 (NCT05017480) [34]; Phase 2a Study (NCT04922021) [36]; Phase 2b Study (NCT03568162) [22]

BNMAs were feasible for all selected efficacy outcomes. The availability of reported endpoints across the included RCTs determined the network structure (Figure 2). All 17 studies provided data for the four prespecified outcomes, EASI‑75, EASI‑90, IGA (or IGA‑AD), and pruritus/itch NRS. Risk of bias assessment revealed limited concerns overall. Four studies [6, 9-11] were rated as having “some concerns”; one study [5] was assessed as “high risk,” and the remaining [8, 12, 19-21, 32-34, 36, 43] were judged to have “low risk” of bias. Every trial included full data and used appropriate blinding, allocation concealment, and randomization techniques (Figure 3). Review-level assessment using ROBIS and network-level evaluation following the ROB-NMA 2025 framework revealed no major concerns regarding study selection, synthesis, transitivity, or inconsistency [66].

Efficacy Endpoints

Among the approved biologic monotherapies (Table 3), dupilumab 300 mg (every two weeks (q2w)) demonstrated strong effectiveness across studies, with placebo‑adjusted responses of 32%-41.5% for EASI‑75, 23%-28% for EASI‑90, 28% for IGA-AD 0/1, and 26%-33.4% for itch NRS (p < 0.0001). Similarly, lebrikizumab 250 mg (q2w) showed robust and consistent efficacy, with placebo‑adjusted improvements of 33.3%-42.0% for EASI‑75, 20.7%-32.6% for EASI‑90, 21.9%-29.7% for IGA-AD 0/1, and 28.3%-42.7% for itch NRS (p < 0.001). In contrast, tralokinumab 300 mg (q2w) produced more modest yet consistent gains, with placebo‑adjusted improvements of 12.1%-22% for EASI‑75 (p ≤ 0.003), 13.7% for EASI‑90, 8.6%-13.8% for IGA-AD 0/1 (p ≤ 0.002), and 9.7%-21.7% for itch NRS (p ≤ 0.001).

**Table 3:** Efficacy outcomes across included randomized trials for atopic dermatitis Abbreviations: APG777, Zumilokibart; EASI-75/90, Eczema Area and Severity Index 75%/90% improvement; IGA-AD, Investigator’s Global Assessment for Atopic Dermatitis (0/1); NA, not available; NCT, National Clinical Trial unique identifier assigned to studies registered on ClinicalTrials.gov; NRS, Numerical Rating Scale; q2w/q4w, every two/four weeks; RR, risk ratio *Approved biologics include an initial loading/induction phase followed by maintenance dosing. For consistency, full dosing regimens (loading and maintenance) are presented in tables, while the manuscript text refers to maintenance doses.

Trial name	Drugs/Dose	Total Patients (n)	EASI-75	Placebo % Adjusted	EASI-90	Placebo % Adjusted	IGA-AD	Placebo % Adjusted	Itch NRS ≥4 pt Responders, n (%)	Placebo % Adjusted
			Responders, n (%)		Responders, n (%)		Responders, n (%)			
Advocate 1 (NCT04146363) [12]	Lebrikizumab: 500 mg at Weeks 0 and 2, followed by 250 mg (q2w)^*^	283	166 (58.8)	42.0 (p<0.001)	108 (38.3)	28.8 (p<0.001)	122 (43.1)	29.7 (p<0.001)	130 (45.9)	32.9 (p<0.001)
	Placebo	141	23 (16.2)		13 (9)		36 (12.7)		18 (13)	
Advocate 2 (NCT04178967) [12]	Lebrikizumab: 500 mg at Weeks 0 and 2, followed by 250 mg (q2w)^*^	281	146 (52.1)	33.3 (p<0.001)	86 (30.7)	20.7 (p<0.001)	93 (33.2)	21.9 (p<0.001)	112 (39.8)	28.3 (p<0.001)
	Placebo	146	26 (18.1)		14 (9.5)		16 (10.8)		17 (11.5)	
Phase 2b Study (NCT03443024) [11]	Lebrikizumab: 500 mg at Weeks 0 and 2, followed by 250 mg (q2w)^*^	75	45 (60.6)	36.3 (p<0.001)	33 (44)	32.6 (p<0.001)	33 (44.6)	29.3 (p=0.002)	53 (70)	42.7 (p<0.001)
	Placebo	52	13 (24.3)		6 (11.4)		8 (15.3)		14 (27.3)	
SOLO-1 (NCT02277743) [6]	Dupilumab: 600 mg loading dose at Week 0, followed by 300 mg (q2w)^*^	223	115 (51)	36 (p<0.001)	80 (36)	28 (p<0.001)	85 (38)	28 (p<0.001)	87 (41)	29 (p<0.001)
	Placebo	224	33 (15)		17 (8)		23 (10)		26 (12)	
SOLO-2 (NCT02277769) [6]	Dupilumab: 600 mg loading dose at Week 0, followed by 300 mg (q2w)^*^	239	103 (44)	32 (p<0.001)	70 (30)	23 (p<0.001)	84 (36)	28 (p<0.001)	81 (36)	26 (p<0.001)
	Placebo	236	28 (12)		17 (7)		20 (8)		21 (10)	
phase 2b study (NCT01859988) 6 [5]	Dupilumab: 600 mg loading dose at Week 0, followed by 300 mg (q2w)^*^	64	34 (53)	41.5 (p<0.0001)	20 (31.2)	27.4 (p<0.0001)	19 (30)	28.0 (p<0.0001)	26 (41)	33.4 (p<0.0001)
	Placebo	61	7 (11.5)		2 (3.8)		1 (2)		5 (8)	
ECZTRA 1 (NCT03131648) [9]	Tralokinumab: 600 mg loading dose, followed by 300 mg (q2w)^*^	603	150 (25.0)	12.1 (p<0.001)	NA	NA	95 (15.8)	8.6	119 (20.0)	9.7
										(p = 0.002)
	Placebo	199	25 (12.7)		NA		14 (7.1)		20 (10.3)	
ECZTRA 2 (NCT03160885) [9]	Tralokinumab: 600 mg loading dose, followed by 300 mg (q2w)^*^	593	196 (33.2)	21.6 (p<0.001)	NA	NA	131 (22.2)	11.1	144 (25.0)	15.6
								(p < 0.001)		(p < 0.001)
	Placebo	201	23 (11.4)		NA		22 (10.9)		19 (9.5)	
ECZTRA 6 (NCT03526861) [8]	Tralokinumab: 600 mg loading dose, followed by 300 mg (q2w)^*^	97	27 (27.8)	22 (p<0.001)	17 (17.5)	13.7 (p=0.002)	17 (17.5)	13.8	24 (25)	21.7 (p<0.001)
								(p= 0.002)		
	Placebo	94	6 (6.4)		4 (4.3)		4 (4.3)		3 (3.3)	
Phase 2b study (NCT05131477) [21]	Amlitelimab 250 mg (q4w)	77	31 (40.3)	29 (p<0.001)	12 (16)	12 (p<0.001)	17 (22.1)	17 (p<0.01)	19 (24.7)	19 (p<0.001)
	Placebo	79	9 (11.4)		3 (4)		4 (5.1)		4 (5.1)	
OX40 (NCT03703102) [19, 20, 23]	Rocatinlimab 150 mg (q4w)	54	23 (44)	33 (p=0.003)	10 (19)	15	10 (19)	17 (p=0.003)	19 (37)	18 (p=0.003)
						(p=0.003)				
	Rocatinlimab 600 mg (q4w)	54	21 (40)	29 (p=0.002)	6 (12)	8	8 (15)	13 (p=0.002)	24 (46)	27 (p=0.002)
						(p=0.002)				
	Rocatinlimab 300 mg (q2w)	55	28 (54)	17 (p<0.001)	19 (37)	33 (p<0.001)	16 (31)	29 (p<0.001)	29 (56)	37 (p<0.001)
	Rocatinlimab 600 mg (q2w)	54	21 (39)	10 (p<0.001)	10 (19)	15 (p<0.001)	10 (19)	16 (p<0.001)	24 (44)	25 (p<0.001)
	Placebo	57	6 (11)		2 (4)		1 (2)		11 (19)	
IGNITE (NCT05398445) [20]	Rocatinlimab 150 mg (q4w)	760	276 (36.3)	23.4 (p<0.001)	NA	NA	145 (19.1)	10.3 (p<0.002)	NA	NA
	Placebo		98 (12.9)		NA		67 (8.8)		NA	
	Rocatinlimab 300 mg (q4w)		321 (42.23)	29.5 (p<0.001)	NA	NA	179 (23.6)	4.9 (p<0.001)	NA	NA
	Placebo		97 (12.73)		NA		142 (18.7)		NA	
REZOLVE-AD (NCT06136741) [43]	Rezpegaldesleukin 24 µg/kg (q2w)	104	44 (42)	25 (p<0.001)	26 (25)	16 (p<0.05)	21 (20)	12 (p<0.05)	44 (42)	26 (p<0.01)
	Rezpegaldesleukin 18 µg/kg (q2w)	106	49 (46)	29 (p<0.001)	19 (18)	9 (p<0.001)	28 (26)	18 (p<0.01)	37 (35)	19 (p<0.05)
	Rezpegaldesleukin 24 µg/kg (q4w)	110	37 (34)	17 (p<0.05)	19 (17)	8 (p<0.05)	21 (19)	11 (p<0.05)	25 (23)	7 (p<0.05)
	Placebo	73	12 (17)		7 (9)		6 (8)		12 (16)	
APEX Phase 2 Part A (NCT06395948) [32]	APG777 720 mg at Weeks 0 and 2, followed by 360 mg at Weeks 4 and 12	82	55 (66.9)	42.3 (p<0.001)	28 (33.9)	19.2 (p<0.05)	29 (34.9)	17.6 (p<0.05)	NA	NA
	Placebo	41	10 (24.6)		6 (14.7)		7 (17.3)		NA	
CBP-201 (NCT05017480) [34]	Rademikibart 300 mg (q2w)	219	128 (58.6)	36 (p<0.001)	73 (33.4)	27	64 (29)	23.1 (p<0.001)	80 (36.6)	26.1
						(p< 0.001)				(p< 0.001)
	Placebo	111	25 (22.6)		7 (6.4)		7 (5.9)		12 (10.5)	
Phase 2a study (NCT04922021) [36]	Temtokibart 450 mg (q2w) (dose per trial arm)	29	12 (41.6)	27.9 (p=0.011)	9 (30.8)	27.3	8 (27.3)	20.3 (p=0.006)	NA	NA
						(p = 0.003)				
	Placebo	29	4 (13.7)		1 (3.5)		2 (7)		NA	
Phase 2b Study (NCT03568162) [22]	Telazorlimab 300 mg (q2w)	76	18 (23.7)	12.4	NA	NA	10 (13.2)	8	6 (7.9)	-2.1
	Telazorlimab 300 mg (q4w)	78	16 (20.5)	9.2	NA	NA	8 (10.3)	5.3	9 (11.5)	1.5
	Telazorlimab 75 mg (q4w)	77	9 (11.7)	0.4	NA	NA	5 (6.5)	1.5	4 (5.2)	-4.8
	Placebo	80	9 (11.3)		NA		4 (5.0)		8 (10.0)	
	Telazorlimab 600 mg (q2w)	75	19 (25.3)	6.4	NA	NA	9 (12.0)	6.6	10 (13.3)	3.8
	Placebo	74	14 (18.9)		NA		4 (5.4)		7 (9.5)	

Among investigational biologic monotherapies and immunomodulators (Table 3), efficacy varies widely across doses and drug classes, with the strongest overall signals observed for zumilokibart, rademikibart, temtokibart, and selected rocatinlimab regimens. Zumilokibart demonstrated one of the most balanced efficacy profiles across endpoints (42.3% EASI‑75, p < 0.001; 19.2% EASI‑90, p < 0.05). Rademikibart 300 mg (q2w) showed strong performance, with 36% EASI‑75 (p < 0.001), 27% EASI‑90 (p < 0.001), and a high itch response of 26.1% (p < 0.001), indicating deep disease and symptom improvement. Temtokibart 450 mg (q2w) also performed competitively, with 27.9% EASI‑75 (p = 0.011), 27.3% EASI-90 (p = 0.003), and 20.3% IGA responders (p = 0.006), making it comparable to leading agents. For rocatinlimab, efficacy varied by dose and schedule, but 300 mg (q2w) stood out with an unusually high 33% EASI‑90 (p < 0.001), 29% IGA (p < 0.001), and the highest itch response (37%, p < 0.001), suggesting that higher‑frequency IL‑31 inhibition may produce greater symptomatic relief. Rezpegaldesleukin had some skin and itch symptom responses, but these waned significantly at the lower dose interval (q4w). Amlitelimab had a moderate level of responses in aggregate (29% EASI 75, p <0.001; 12% EASI 90, p <0.001). On the other hand, telazorlimab had a persistent poor response for all endpoints, with EASI-75 less than 10%. In aggregate, the best responsive investigational biologics for both skin clearance and itch symptom relief were zumilokibart, rademikibart, temtokibart, and rocatinlimab at dosages of 300 mg (q2w), with telazorlimab demonstrated to be the least responsive among the tested agents.

Pooled Efficacy Analysis

A combined analysis was performed (Table 4) to enhance sensitivity and ensure harmonized and comparable estimates of efficacy for biologics monotherapies. The investigational biologics zumilokibart (360 mg) had high levels of efficacy, with an EASI-75 response rate of 42.3% cumulatively for all treatments, but a surprisingly lower response rate for stricter endpoints (EASI-90, 19.2%; IGA-AD 0/1, 17.6%), indicating strong moderate responses but relatively low rates of near-complete remission of the disease. Rademikibart 300 mg (q2w) had strong and well-balanced responses, with response rates of 36% EASI-75, 27% EASI-90, 23.1% IGA-AD 0/1, and 26.1% itch NRS response. The degree of deep response was best with rocatinlimab 300 mg (q2w), with response rates of 33% EASI-90, 29% IGA-AD 0/1, and 37% ttch NRS response, which were the highest in their respective categories and suggested greater sensitivity for complete disease control. The other candidates with some degree of activity were rezpegaldesleukin with a response rate of approximately 40% EASI-75 and 26% itch response, temtokibart with responses of 27.9% EASI-75 and 27.3% EASI-90, and amlitelimab of 29% EASI-75 response rates, but telazorlimab regimens varied widely, with generally lower responses and some doses showing minimal improvement or worsening of itch symptoms.

**Table 4:** Pooled Efficacy Estimates for All Outcomes Abbreviations: APG777, Zumilokibart; EASI-75/90, Eczema Area and Severity Index 75%/90% improvement; IGA-AD, Investigator’s Global Assessment for Atopic Dermatitis; NRS, Numerical Rating Scale; q2w/q4w, every two/four weeks; mg, milligrams; µg/kg, micrograms per kilogram; NA, not available *Approved biologics include an initial loading/induction phase followed by maintenance dosing. For consistency, full dosing regimens (loading and maintenance) are presented in tables, while the manuscript text refers to maintenance doses.

Drugs/Dose	Total Patients (n)	EASI-75	EASI-75	Placebo % Adjusted	EASI-90	EASI-90	Placebo % Adjusted	IGA-AD	IGA-AD	Placebo % Adjusted	Itch NRS ≥4 pt Responders (n)	Itch NRS ≥4 pt Responders, n (%)	Placebo % Adjusted
		Responders (n)	Responders (%)		Responders (n)	Responders (%)		Responders (n)	Responders (%)				
Lebrikizumab: 500 mg at Weeks 0 and 2, followed by 250 mg (q2w)^*^ [11, 12]	639	357	55.87	34.9	227	35.52	25.8	248	38.81	21.1	295	46.17	31.7
Placebo	339	71	20.94		33	9.73		60	17.7		49	14.45	
Dupilumab: 600 mg loading dose at Week 0, followed by 300 mg (q2w)^*^ [5, 6]	526	252	47.91	37.93	170	32.32	25.41	188	35.74	27.3	194	36.88	26.9
Placebo	521	52	9.98		36	6.91		44	8.45		52	9.98	
Tralokinumab: 600 mg loading dose, followed by 300 mg (q2w)^*^ [8] [9]	1293	373	28.85	17.9	17	17.53	13.7	243	18.79	10.7	287	22.2	13.7
Placebo	494	54	10.93		4	4.26		40	8.1		42	8.5	
Amlitelimab 250 mg (q4w) [21]	77	31	40.26	29	12	15.58	12	17	22.08	17	19	24.68	19
Placebo	79	9	11.39		3	3.8		4	5.06		4	5.06	
Rocatinlimab 150 mg (q4w) (dose per trial arm) [19, 20, 23]	54	23	42.59	33	10	18.52	15	10	18.52	17	19	35.19	18
Rocatinlimab 600 mg (q4w) (dose per trial arm) [19, 20, 23]	54	21	38.89	29	6	11.11	8	8	14.81	13	24	44.44	27
Rocatinlimab 300 mg (q2w) (dose per trial arm) [19, 20, 23]	55	28	50.91	17	19	34.55	33	16	29.09	29	29	52.73	37
Rocatinlimab 600 mg (q2w) (dose per trial arm) [19, 20, 23]	54	21	38.89	10	10	18.52	15	10	18.52	16	24	44.44	25
Placebo	57	6	10.53		2	3.51		1	1.75		11	19.3	
Rocatinlimab 150 mg (q4w) (dose per trial arm) [20]	760	276	36.32	23.4	NA	NA	NA	145	19.08	10.3	NA	NA	NA
Placebo	760	98	12.89		NA	NA		67	8.82		NA	NA	
Rocatinlimab 300 mg (q4w) (dose per trial arm) [20]	760	321	42.24	29.5	NA	NA	NA	179	23.55	4.9	NA	NA	NA
Placebo	760	97	12.76		NA	NA		142	18.68		NA	NA	
Rezpegaldesleukin 24 ug/kg (q2w) [43]	104	44	42.31	25	26	25	16	21	20.19	12	44	42.31	26
Rezpegaldesleukin 18 ug/kg (q2w) [43]	106	49	46.23	29	19	17.92	9	28	26.42	18	37	34.91	19
Rezpegaldesleukin 24 ug/kg (q4w) [43]	110	37	33.64	17	19	17.27	8	21	19.09	11	25	22.73	7
Placebo	73	12	16.44		7	9.59		6	8.22		12	16.44	
APG777 720 mg at Weeks 0 and 2, followed by 360 mg at Weeks 4 and 12 [32]	82	55	67.07	42.3	28	34.15	19.2	29	35.37	17.6	NA	NA	NA
Placebo	41	10	24.39		6	14.63		7	17.07		NA	NA	
Rademikibart 300 mg (q2w) [34]	219	128	58.45	36	73	33.33	27	64	29.22	23.1	20	9.13	26.1
Placebo	111	25	22.52		7	6.31		7	6.31		12	10.81	
Temtokibart 450 mg (q2w) (dose per trial arm) [36]	29	12	41.38	27.9	9	31.03	27.3	8	27.59	20.3	NA	NA	NA
Placebo	29	4	13.79		1	3.45		2	6.9		NA	NA	
Telazorlimab 300 mg (q2w) [22]	76	18	23.68	12.4	NA	NA	NA	10	13.16	8	6	7.89	NA2.1
Telazorlimab 300 mg (q4w) [22]	78	16	20.51	9.2	NA	NA	NA	8	10.26	5.3	9	11.54	1.5
Telazorlimab 75 mg (q4w) [22]	77	9	11.69	0.4	NA	NA	NA	5	6.49	1.5	7	9.09	NA4.8
Placebo	80	9	11.25		NA	NA		4	5		8	10	
Telazorlimab 600 mg (q2w) [22]	75	19	25.33	6.4	NA	NA	NA	9	12	6.6	10	13.33	3.8
Placebo	74	14	18.92		NA	NA	5.41	4	5.41		7	9.46	

Of the approved drugs (Table 4), dupilumab 300 mg (q2w) showed the strongest and most consistent efficacy for 37.9% EASI-75, 25.4% EASI-90, 27.3% IGA-AD 0/1, and 26.9% itching improvement. Lebrikizumab 250 mg (q2w) showed comparable efficacy of 34.9% EASI-75, 25.8% EASI-90, and especially significant itching improvement of 31.7%, although slightly lower IGA-AD 0/1 response at 21.1%. Tralokinumab 300 mg (q2w) showed the poorest responses among approved biologics, with all values showing significantly lower efficacy: 17.9% EASI-75, 13.7% EASI-90, 10.7% IGA-AD 0/1, and 13% itching improvement.

Among approved drugs, dupilumab showed the most consistent efficacy across endpoints, followed by lebrikizumab, with more modest responses observed for tralokinumab. Of the investigational biologics tested, rocatinlimab 300 mg (q2w) had the best deep response profile with regard to the highest EASI-90, IGA-AD 0/1, and pruritus improvement rates, although the best EASI-75 response was achieved with zumilokibart. Some investigational agents were found to have superior outcomes when compared with approved therapies on certain outcomes and may achieve better symptom control depending on the results of larger confirmatory studies.

Bayesian NMA

**Figure 4:**
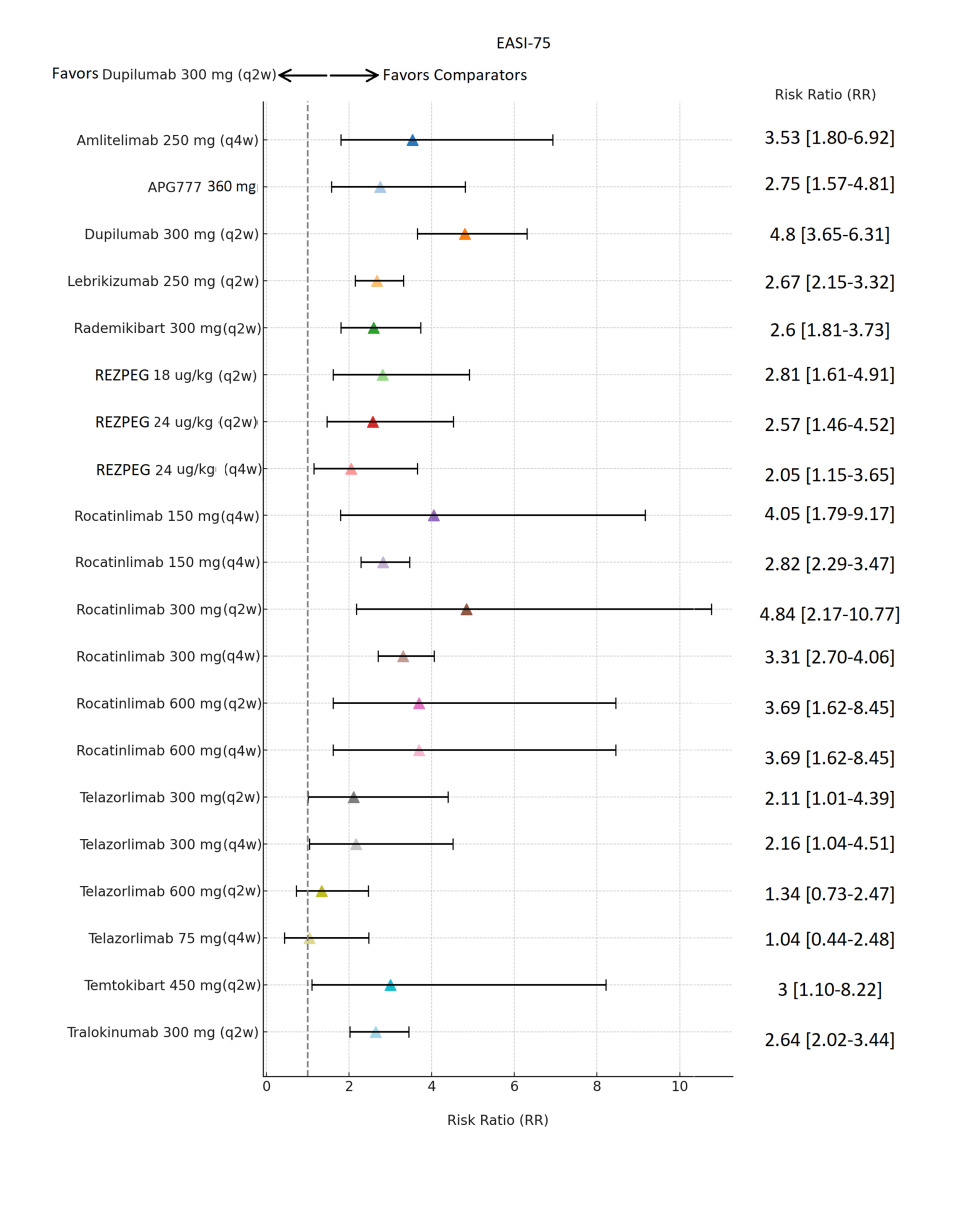
Network meta-analysis: Eczema Area and Severity Index 75% improvement (EASI-75) response Forest plot displaying risk ratios with 95% credible intervals comparing each treatment to placebo for achieving EASI-75. Results are based on maintenance dosing regimens; approved biologics included dupilumab 300 mg (q2w) following a 600 mg loading dose (LD), lebrikizumab 250 mg (q2w) following a 500 mg LD at Weeks 0 and 2, and tralokinumab 300 mg (q2w)) following a 600 mg LD. Abbreviations: APG777, Zumilokibart; REZPEG; Rezpegaldesleukin. Advocate 1 (NCT04146363) [12]; Advocate 2 (NCT04178967) [12]; Phase 2b Study (NCT03443024) [11]; SOLO-1 (NCT02277743) [6]; SOLO-2 (NCT02277769) [6]; Phase 2b Study (NCT01859988) [5]; ECZTRA 1 (NCT03131648) [9]; ECZTRA 2 (NCT03160885) [9]; ECZTRA 6 (NCT03526861) [8]; Phase 2b Study (NCT05131477) [21]; OX40 (NCT03703102) [19, 20, 23]; IGNITE (NCT05398445) [20]; REZOLVE-AD (NCT06136741) [43]; APEX Phase 2 Part A (NCT06395948) [32]; CBP-201 (NCT05017480) [34]; Phase 2a Study (NCT04922021) [36]; Phase 2b Study (NCT03568162) [22]; q2w, every two weeks; q4w, every four weeks

**Figure 5:**
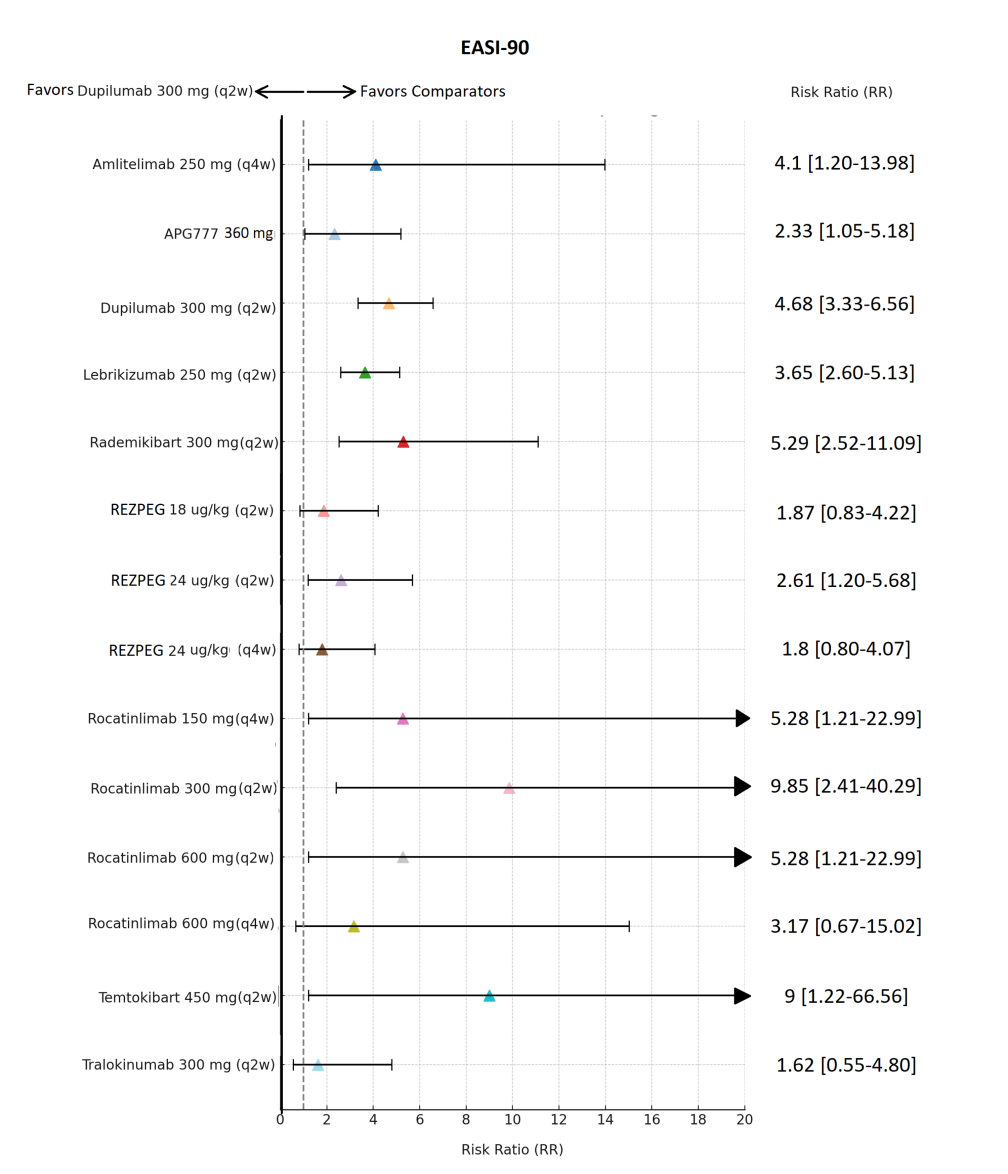
Network meta-analysis: Eczema Area and Severity Index 90% improvement (EASI-90) response Forest plot presenting risk ratios with 95% credible intervals for each therapy versus placebo in achieving EASI-90 response. Effect estimates correspond to maintenance doses (dupilumab 300 mg (q2w), lebrikizumab 250 mg (q2w), tralokinumab 300 mg (q2w) administered after protocol-specified loading doses. Investigational agents followed trial-specific induction regimens. Abbreviations: APG777, Zumilokibart; REZPEG; Rezpegaldesleukin. Advocate 1 (NCT04146363) [12]; Advocate 2 (NCT04178967) [12]; Phase 2b Study (NCT03443024) [11]; SOLO-1 (NCT02277743) [6]; SOLO-2 (NCT02277769) [6]; Phase 2b Study (NCT01859988) [5]; ECZTRA 1 (NCT03131648) [9]; ECZTRA 2 (NCT03160885) [9]; ECZTRA 6 (NCT03526861) [8]; Phase 2b Study (NCT05131477) [21]; OX40 (NCT03703102) [19, 20, 23]; IGNITE (NCT05398445) [20]; REZOLVE-AD (NCT06136741) [43]; APEX Phase 2 Part A (NCT06395948) [32]; CBP-201 (NCT05017480) [34]; Phase 2a Study (NCT04922021) [36]; Phase 2b Study (NCT03568162) [22]; q2w, every two weeks; q4w, every four weeks

**Figure 6:**
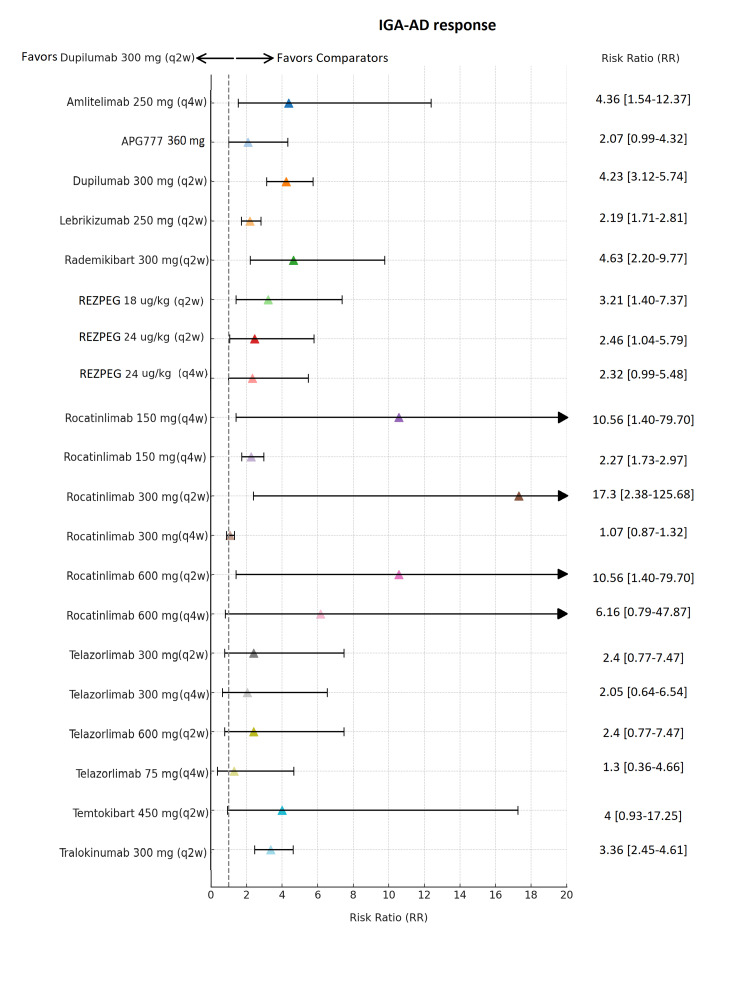
Network meta-analysis: Investigator’s Global Assessment for Atopic Dermatitis (IGA‑AD) response Forest plot illustrating risk ratios with 95% credible intervals comparing treatments to placebo for achieving a validated IGA‑AD 0/1 response. Approved biologics were administered with loading doses prior to maintenance therapy (dupilumab 600 mg → 300 mg (q2w); lebrikizumab 500 mg at Weeks 0 and 2 → 250 mg (q2w); tralokinumab 600 mg → 300 mg (q2w)). Abbreviations: APG777, Zumilokibart; REZPEG; Rezpegaldesleukin. Advocate 1 (NCT04146363) [12]; Advocate 2 (NCT04178967) [12]; Phase 2b Study (NCT03443024) [11]; SOLO-1 (NCT02277743) [6]; SOLO-2 (NCT02277769) [6]; Phase 2b Study (NCT01859988) [5]; ECZTRA 1 (NCT03131648) [9]; ECZTRA 2 (NCT03160885) [9]; ECZTRA 6 (NCT03526861) [8]; Phase 2b Study (NCT05131477) [21]; OX40 (NCT03703102) [19, 20, 23]; IGNITE (NCT05398445) [20]; REZOLVE-AD (NCT06136741) [43]; APEX Phase 2 Part A (NCT06395948) [32]; CBP-201 (NCT05017480) [34]; Phase 2a Study (NCT04922021) [36]; Phase 2b Study (NCT03568162) [22]; q2w, every two weeks; q4w, every four weeks

**Figure 7:**
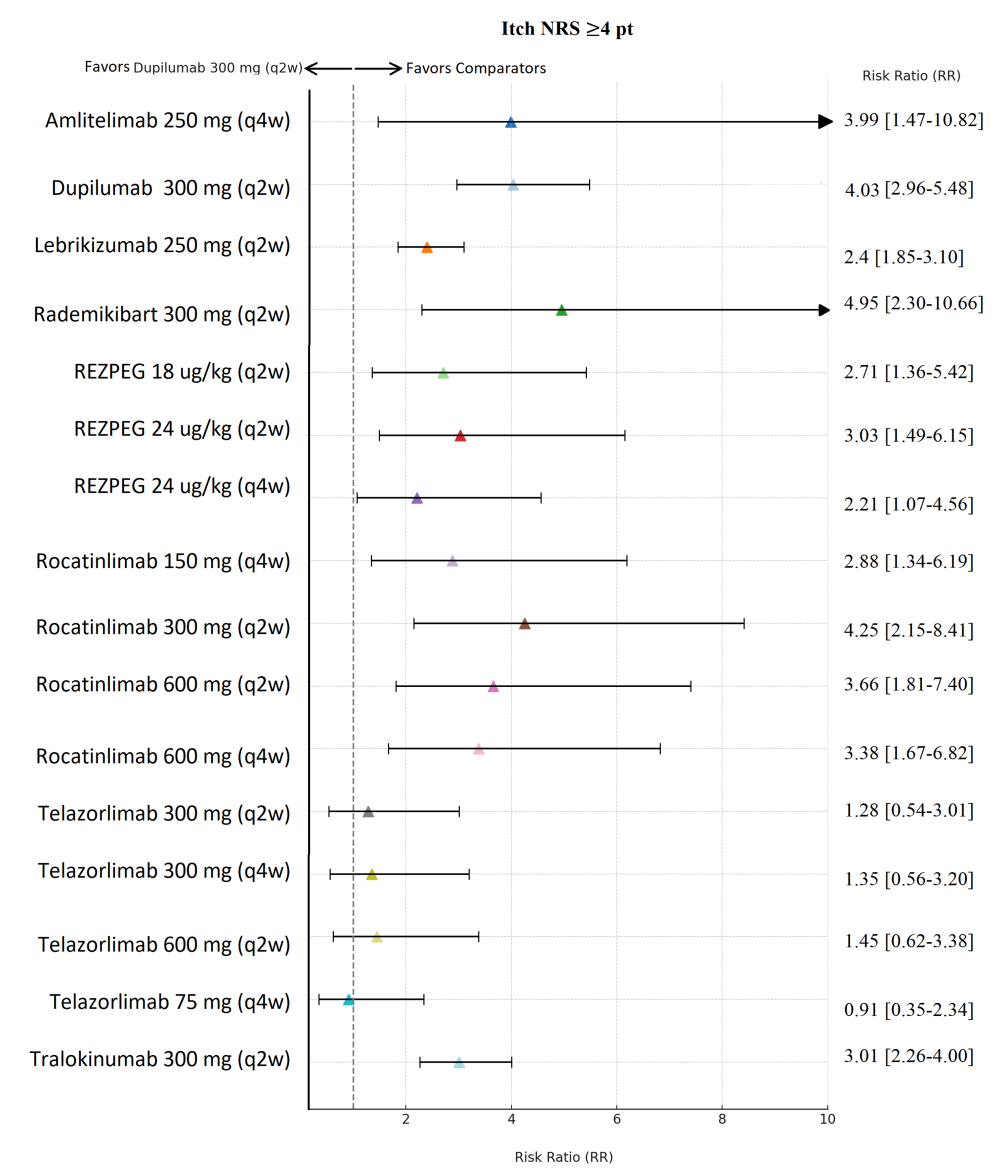
Network meta-analysis: Itch Numeric Rating Scale (NRS) ≥4-point improvement. Forest plot summarizing risk ratios with 95% credible intervals for clinically meaningful itch reduction (≥4-point NRS improvement) versus placebo. Comparisons reflect maintenance dosing regimens following loading doses for approved biologics (dupilumab, lebrikizumab, tralokinumab), while investigational agents were administered per study-specific induction protocols. Abbreviation: REZPEG; Rezpegaldesleukin. Advocate 1 (NCT04146363) [12]; Advocate 2 (NCT04178967) [12]; Phase 2b Study (NCT03443024) [11]; SOLO-1 (NCT02277743) [6]; SOLO-2 (NCT02277769) [6]; Phase 2b Study (NCT01859988) [5]; ECZTRA 1 (NCT03131648) [9]; ECZTRA 2 (NCT03160885) [9]; ECZTRA 6 (NCT03526861) [8]; Phase 2b Study (NCT05131477) [21]; OX40 (NCT03703102) [19, 20, 23]; IGNITE (NCT05398445) [20]; REZOLVE-AD (NCT06136741) [43]; APEX Phase 2 Part A (NCT06395948) [32]; CBP-201 (NCT05017480) [34]; Phase 2a Study (NCT04922021) [36]; Phase 2b Study (NCT03568162) [22]; q2w, every two weeks; q4w, every four weeks

The BNMA compared the relative efficacy of 10 biologic monotherapies: lebrikizumab 250 mg, rocatinlimab 300/600 mg, dupilumab 300 mg, rezpegaldesleukin 24µg/kg or 18µg/kg, amlitelimab 250 mg, tralokinumab 300mg, telazorlimab 75/300/600 mg, temtokibart 450 mg, rademikibart 300mg, and zumilokibart 360 mg (Figures 4-7) [5-11, 13-15, 18, 20-23]. The efficacy endpoints explored in the meta-analysis included EASI-75, EASI-90, IGA AD 0/1, and NRS ≥4- point improvement in itch NRS. The forest plots in the meta-analysis (Figures 4-7) illustrate the relative risk of clinical response for each biologic compared with placebo, with triangles representing point estimates and horizontal lines indicating 95% uncertainty intervals.

In the analysis of EASI-75 in Figure 4, dupilumab 300 mg (q2w) continued to be a major comparator with an RR of 4.8 (3.65, 6.31), being closely rivaled by rocatinlimab 300 mg (q2w), showing the largest size effect in this model with 4.84 (2.17, 10.77), followed by rocatinlimab 150 mg (q4w) in a small cohort showing 4.05 (1.79, 9.17). Other drugs showing prominent efficacy were amlitelimab 250 mg (q4w) at 3.53 (1.80, 6.92), zumilokibart (360 mg) at 2.75 (1.57, 4.81), lebrikizumab 250 mg (q2w) at 2.67 (2.15, 3.32), Rademikibart 300 mg (q2w) at 2.6 (1.81, 3.73), and rezpegaldesleukin across the dose regimen with risk ratios varying between 2.81 (1.61, 2.81(1.61,4.91) and 2.05 (1.15,3.65). In the EASI‑90 analysis (Figure 5), several emerging agents showed higher numerical RRs than dupilumab, but most were accompanied by very wide CrIs, limiting the certainty of their effects. Dupilumab 300 mg (q2w) demonstrated strong and reliable performance, with an RR of 4.68 (95% Crl, 3.33-6.56), combining high efficacy with excellent precision and making it one of the most stable and trustworthy treatments. Rocatinlimab 300 mg (q2w) achieved the highest point estimate (RR 9.85; 95% Crl, 2.41-40.29), and temtokibart 450 mg (q2w) showed a similarly high estimate (RR 9.0; 95% Crl, 1.22-66.56), but the extremely wide CrIs indicate major uncertainty and low precision; therefore, these results cannot be interpreted as definitively superior to dupilumab. Other agents such as amlitelimab (RR 4.1; 95% Crl, 1.20-13.98), zumilokibart (RR 2.33; 95% Crl, 1.05-5.18), rademikibart (RR 5.29; 95% Crl, 2.52-11.09), and lebrikizumab (RR 3.65; 95% Crl, 2.60-5.13) demonstrated varying degrees of efficacy, but none combined high effect with tight precision to the extent observed with dupilumab.

Across all therapies, dupilumab 300 mg (q2w) demonstrated the strongest and most reliable IGA‑AD response (Figure 6), with an RR of 4.23 (95% Crl, 3.12-5.74) and a narrow Crl, indicating consistent and reproducible efficacy across patients. Although some agents reported higher numerical RRs, such as rocatinlimab 300 mg (q2w) (RR 17.3; 95% Crl, 2.38-125.68) and rocatinlimab 150 mg (q4w) (RR 10.56; 95% Crl, 1.40-79.70), their extremely wide Crls reflect substantial uncertainty, making the true effect difficult to estimate with confidence. Moderate-effect treatments such as amlitelimab (RR 4.36; 95% Crl, 1.54-12.37), rademikibart (RR 4.63; 95% Crl, 2.20-9.77), and tralokinumab (RR 3.36; 95% Crl, 2.45-4.61) contributed positively but remained less consistent than dupilumab. Lower and more variable results were observed with rezpegaldesleukin (RR 2.32-3.21; 95% Crl, 0.99-7.37) and telazorlimab (RR 1.3-2.4; 95% Crl, 0.36-7.47).

Dupilumab 300 mg (q2w) demonstrated one of the strongest and most precise itch responses, with an RR of 4.03 (95% Crl, 2.96-5.48), indicating a robust and reliable reduction in itch severity (Figure 7). Although rademikibart 300 mg (q2w) (RR 4.95; 95% CrI, 2.30-10.66) and amlitelimab 250 mg (q4w) (RR 3.99; 95% CrI, 1.47-10.82) showed numerically high effects, their wide CrIs reflect substantial uncertainty, reducing confidence in their superiority. Rezpegaldesleukin across doses demonstrated moderate efficacy (RR 2.21-3.03) with reasonably consistent CrIs, while rocatinlimab produced mixed results (RRs 2.88-4.25) but again with wide CrIs, indicating variability. Telazorlimab yielded lower and imprecise effects (RR 0.91-1.45; 95% Crl, 0.35-3.38), suggesting weak or uncertain benefit.

Across all outcomes (EASI‑90, IGA‑AD, and itch NRS ≥4), dupilumab 300 mg (q2w) consistently provided the strongest and most reliable performance, combining high efficacy with narrow, precise CIs. Although several newer agents reported higher point estimates, their effects were undermined by wide CrIs and low precision. Collectively, dupilumab remains the most dependable and clinically robust treatment across endpoints.

SUCRA Probability Analyses

**Figure 8:**
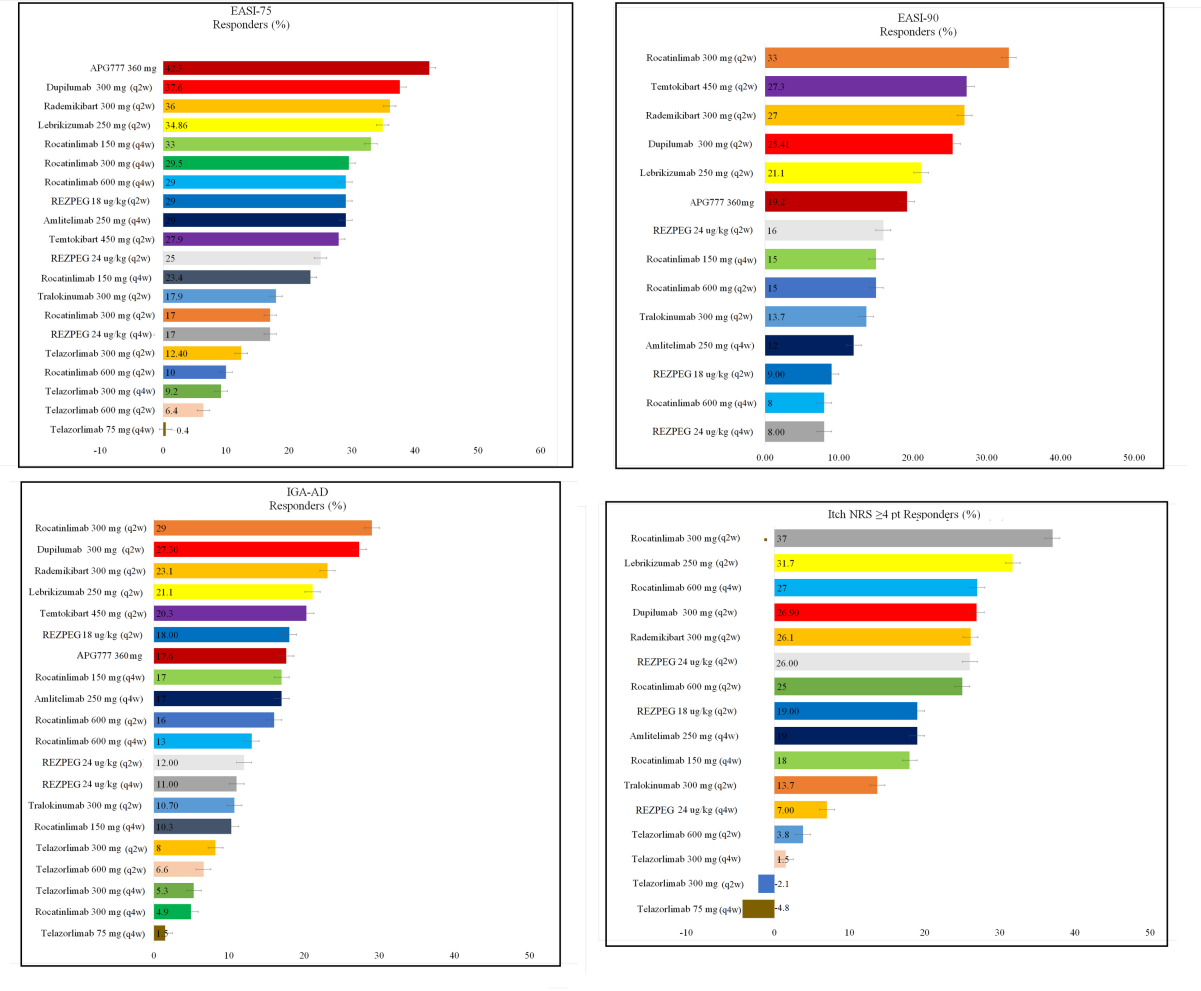
Surface Under the Cumulative Ranking curve (SUCRA) scores for all treatments. Bar charts displaying SUCRA values ranking each treatment’s relative efficacy across key clinical endpoints. Higher SUCRA scores indicate a greater probability of being among the most effective therapies. Abbreviations: APG777, Zumilokibart; REZPEG; Rezpegaldesleukin.  Advocate 1 (NCT04146363) [12]; Advocate 2 (NCT04178967) [12]; Phase 2b Study (NCT03443024) [11]; SOLO-1 (NCT02277743) [6]; SOLO-2 (NCT02277769) [6]; Phase 2b Study (NCT01859988) [5]; ECZTRA 1 (NCT03131648) [9]; ECZTRA 2 (NCT03160885) [9]; ECZTRA 6 (NCT03526861) [8]; Phase 2b Study (NCT05131477) [21]; OX40 (NCT03703102) [19, 20, 23]; IGNITE (NCT05398445) [20]; REZOLVE-AD (NCT06136741) [43]; APEX Phase 2 Part A (NCT06395948) [32]; CBP-201 (NCT05017480) [34]; Phase 2a Study (NCT04922021) [36]; Phase 2b Study (NCT03568162) [22]; EASI‑75, Eczema Area and Severity Index 75% improvement; EASI‑90, Eczema Area and Severity Index 90% improvement; IGA-AD, Investigator’s Global Assessment for Atopic Dermatitis, NRS, Numerical Rating Scale; q2w, every two weeks; q4w, every four weeks

The SUCRA‑equivalent (P‑score) analysis across all evaluated endpoints (Figure 8 and Table 5), including EASI‑75, EASI‑90, IGA‑AD 0/1 response, and ≥4‑point improvement in itch NRS, demonstrated a consistent and reproducible efficacy hierarchy among biologic agents for AD. Dupilumab 300 mg (q2w) showed the highest probability of being the best overall treatment, followed closely by rocatinlimab 300 mg (q2w); both combined strong clinical responses with consistent performance across outcomes. Rademikibart 300 mg (q2w), lebrikizumab 250 mg (q2w), and temtokibart 450 mg (q2w) formed the next tier, demonstrating substantial efficacy but with slightly less uniformity across endpoints. Mid-range probabilities were observed with rezpegaldesleukin, zumilokibart, and tralokinumab, which performed moderately but did not consistently rank near the top. In contrast, telazorlimab regimens showed the lowest SUCRA probabilities, reflecting weaker and less consistent outcomes across all measures. Taken together, the SUCRA analysis confirmed that dupilumab and rocatinlimab offered the highest probability of superior efficacy across endpoints.

**Table 5:** Surface Under the Cumulative Ranking (SUCRA) Scores for All Treatments Across Key Clinical Endpoints Abbreviations: APG777, Zumilokibart; SUCRA, Surface Under the Cumulative Ranking; mg, milligrams; q2w, every two weeks; q4w, every four weeks; µg/kg, micrograms per kilogram. *Approved biologics include an initial loading/induction phase followed by maintenance dosing. For consistency, full dosing regimens (loading and maintenance) are presented in tables, while the manuscript text refers to maintenance doses. Advocate 1 (NCT04146363) [12]; Advocate 2 (NCT04178967) [12]; Phase 2b Study (NCT03443024) [11]; SOLO-1 (NCT02277743) [6]; SOLO-2 (NCT02277769) [6]; Phase 2b Study (NCT01859988) [5]; ECZTRA 1 (NCT03131648) [9]; ECZTRA 2 (NCT03160885) [9]; ECZTRA 6 (NCT03526861) [8]; Phase 2b Study (NCT05131477) [21]; OX40 (NCT03703102) [19, 20, 23]; IGNITE (NCT05398445) [20]; REZOLVE-AD (NCT06136741) [43]; APEX Phase 2 Part A (NCT06395948) [32]; CBP-201 (NCT05017480) [34]; Phase 2a Study (NCT04922021) [36]; Phase 2b Study (NCT03568162) [22]

Rank	Treatment	SUCRA (%)	Interpretation
1	Dupilumab: 600 mg loading dose at Week 0, followed by 300 mg (q2w)^*^	96%	Best overall performer; highest responder rates across multiple outcomes
2	Rocatinlimab 300 mg (q2w) (dose per trial arm)	90%	Strong, consistent performance with high precision
3	Rademikibart 300 mg (q2w)	82%	High efficacy; slightly more variability than top two
4	Lebrikizumab: 500 mg at Weeks 0 and 2, followed by 250 mg (q2w)^*^	75%	Stable, reliable responses across outcomes
5	Temtokibart 450 mg (q2w) (dose per trial arm)	70%	Strong in EASI-90 but inconsistent across others
6	Amlitelimab 250 mg (q4w)	65%	Good responses but wider uncertainty
7	APG777 720 mg at Weeks 0 and 2, followed by 360 mg at Weeks 4 and 12	62%	Moderate but consistent performance
8	Rezpegaldesleukin 24 µg/kg (q2w)	55%	Moderate responses; not top-tier
9	Rezpegaldesleukin 18 µg/kg (q2w)	50%	Mid-range performance
10	Tralokinumab: 600 mg loading dose, followed by 300 mg (q2w)^*^	48%	Reasonable but not competitive with top group
11	Rocatinlimab 150 mg (q4w) (dose per trial arm)	45%	Lower and less consistent than the 300 mg regimen
12	Rezpegaldesleukin 24 µg/kg (q4w)	40%	Inconsistent performance across outcomes
13	Rocatinlimab 600 mg (q2w) (dose per trial arm)	35%	Moderate efficacy with less consistency
14	Rocatinlimab 600 mg (q4w) (dose per trial arm)	30%	Lower and inconsistent results compared to other doses
15	Telazorlimab 300 mg (q2w)	25%	Lower responder rates
16	Telazorlimab 300 mg (q4w)	20%	Weak performance
17	Telazorlimab 600 mg (q2w)	12%	Near the bottom across outcomes
18	Telazorlimab 75 mg (q4w)	8%	Lowest performer across all outcomes

Pairwise Comparisons Using Heat Maps

**Figure 9:**
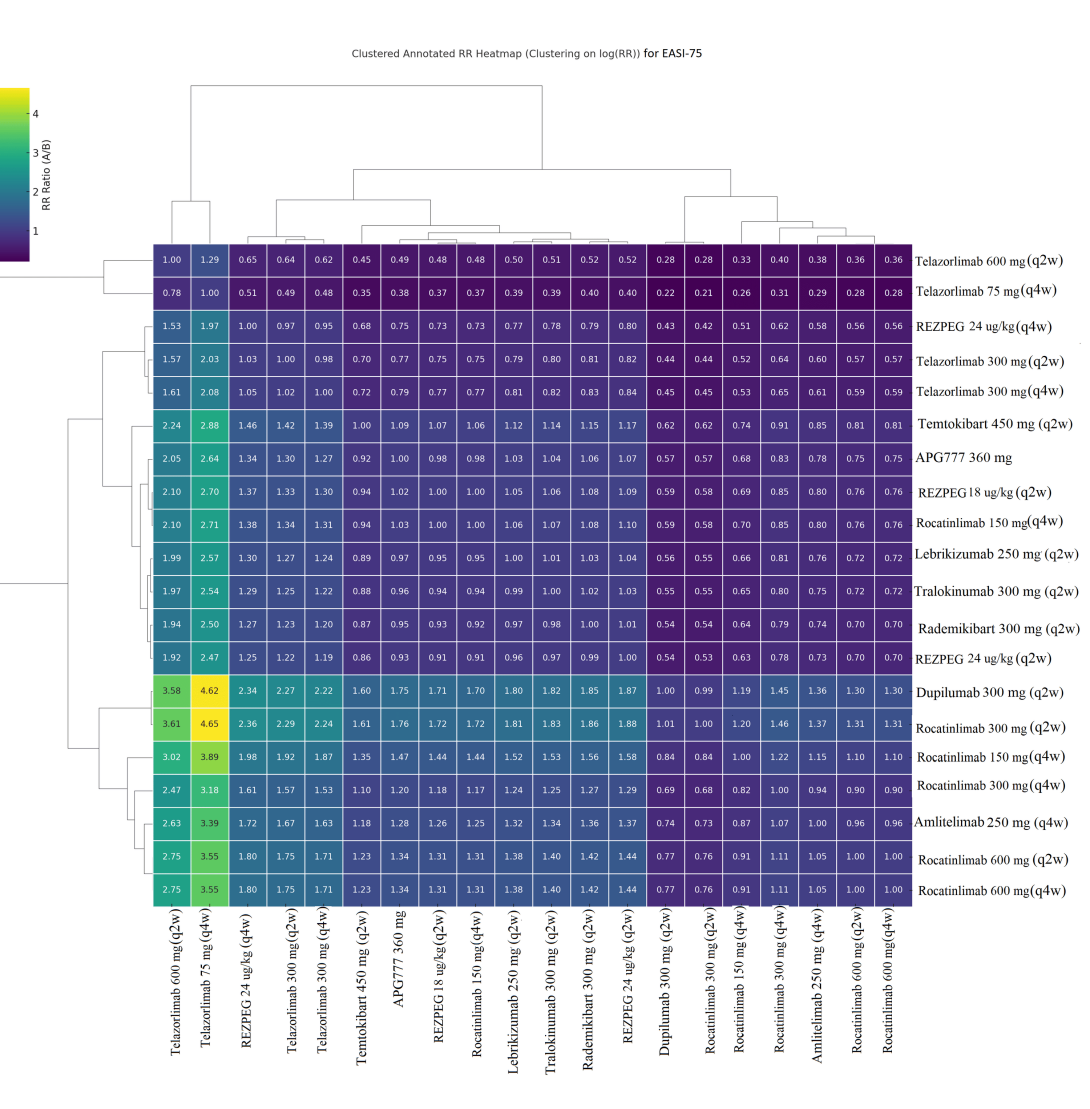
Clustered heatmap of pairwise risk ratios (RRs) for Eczema Area and Severity Index 75% improvement (EASI-75) Clustered heatmap showing pairwise RRs for EASI-75 across all treatments. Hierarchical clustering groups regimens by similarity in relative efficacy. Treatment labels represent maintenance doses, following loading doses (LDs) for approved biologics (dupilumab 600 mg LD → 300 mg (q2w); lebrikizumab 500 mg LD at Weeks 0 and 2 → 250 mg (q2w); tralokinumab 600 mg LD → 300 mg (q2w)) and trial-specific induction regimens for investigational therapies. Abbreviations: APG777, Zumilokibart; REZPEG; Rezpegaldesleukin. Advocate 1 (NCT04146363) [12]; Advocate 2 (NCT04178967) [12]; Phase 2b Study (NCT03443024) [11]; SOLO-1 (NCT02277743) [6]; SOLO-2 (NCT02277769) [6]; Phase 2b Study (NCT01859988) [5]; ECZTRA 1 (NCT03131648) [9]; ECZTRA 2 (NCT03160885) [9]; ECZTRA 6 (NCT03526861) [8]; Phase 2b Study (NCT05131477) [21]; OX40 (NCT03703102) [19, 20, 23]; IGNITE (NCT05398445) [20]; REZOLVE-AD (NCT06136741) [43]; APEX Phase 2 Part A (NCT06395948) [32]; CBP-201 (NCT05017480) [34]; Phase 2a Study (NCT04922021) [36]; Phase 2b Study (NCT03568162) [22]; q2w, every two weeks; q4w, every four weeks

**Figure 10:**
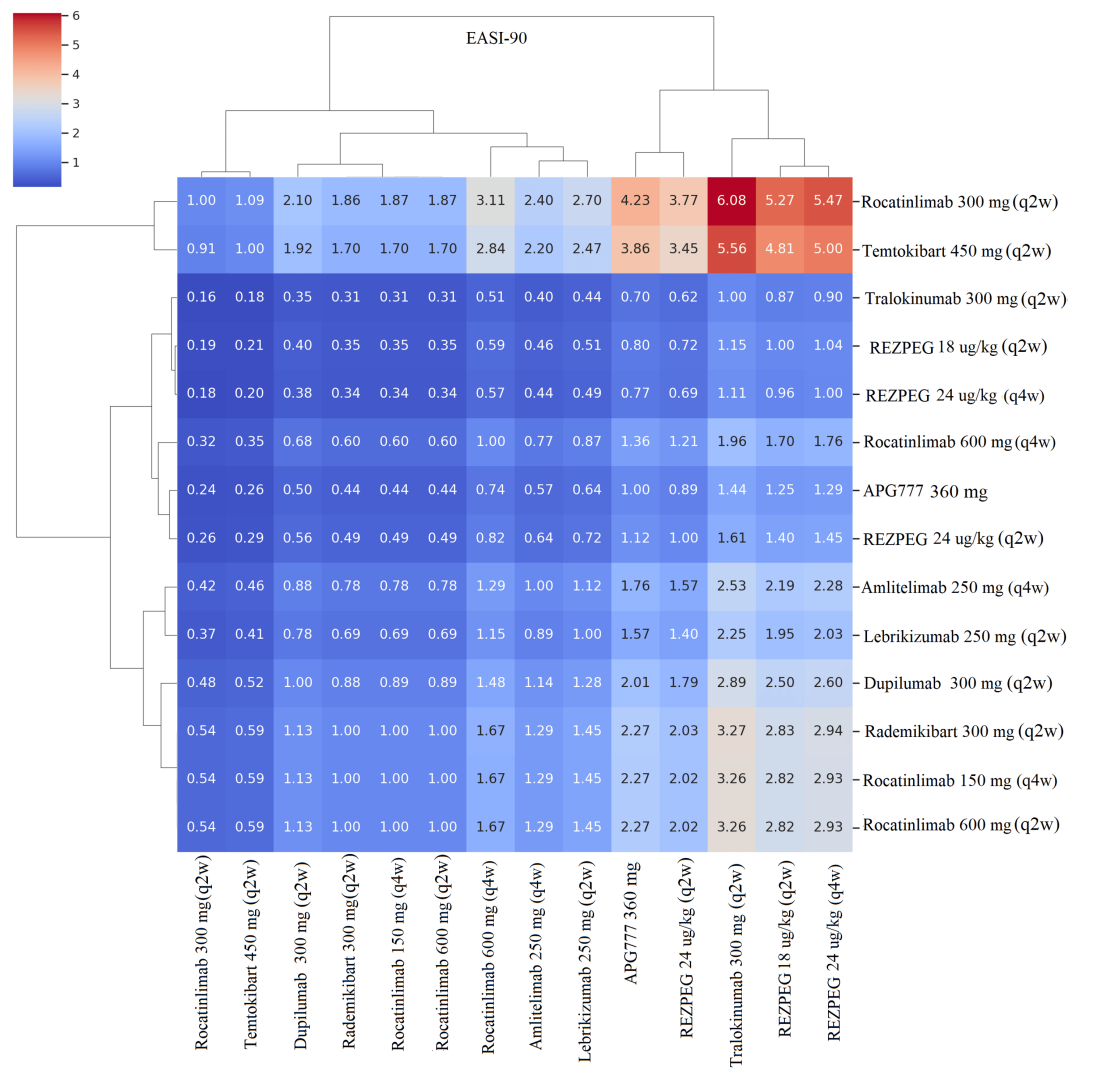
Clustered heatmap of pairwise risk ratios (RRs) for Eczema Area and Severity Index 90% improvement (EASI-90) Heatmap displaying pairwise RRs for EASI-90 with hierarchical clustering. Warmer colors denote higher relative efficacy compared with comparator treatments. All comparisons are based on maintenance dosing regimens, preceded by loading doses for approved biologics and protocol-defined induction schedules for investigational agents. Abbreviations: APG777, Zumilokibart; REZPEG; Rezpegaldesleukin. Advocate 1 (NCT04146363) [12]; Advocate 2 (NCT04178967) [12]; Phase 2b Study (NCT03443024) [11]; SOLO-1 (NCT02277743) [6]; SOLO-2 (NCT02277769) [6]; Phase 2b Study (NCT01859988) [5]; ECZTRA 1 (NCT03131648) [9]; ECZTRA 2 (NCT03160885) [9]; ECZTRA 6 (NCT03526861) [8]; Phase 2b Study (NCT05131477) [21]; OX40 (NCT03703102) [19, 20, 23]; IGNITE (NCT05398445) [20]; REZOLVE-AD (NCT06136741) [43]; APEX Phase 2 Part A (NCT06395948) [32]; CBP-201 (NCT05017480) [34]; Phase 2a Study (NCT04922021) [36]; Phase 2b Study (NCT03568162) [22]; q2w, every two weeks; q4w, every four weeks

**Figure 11:**
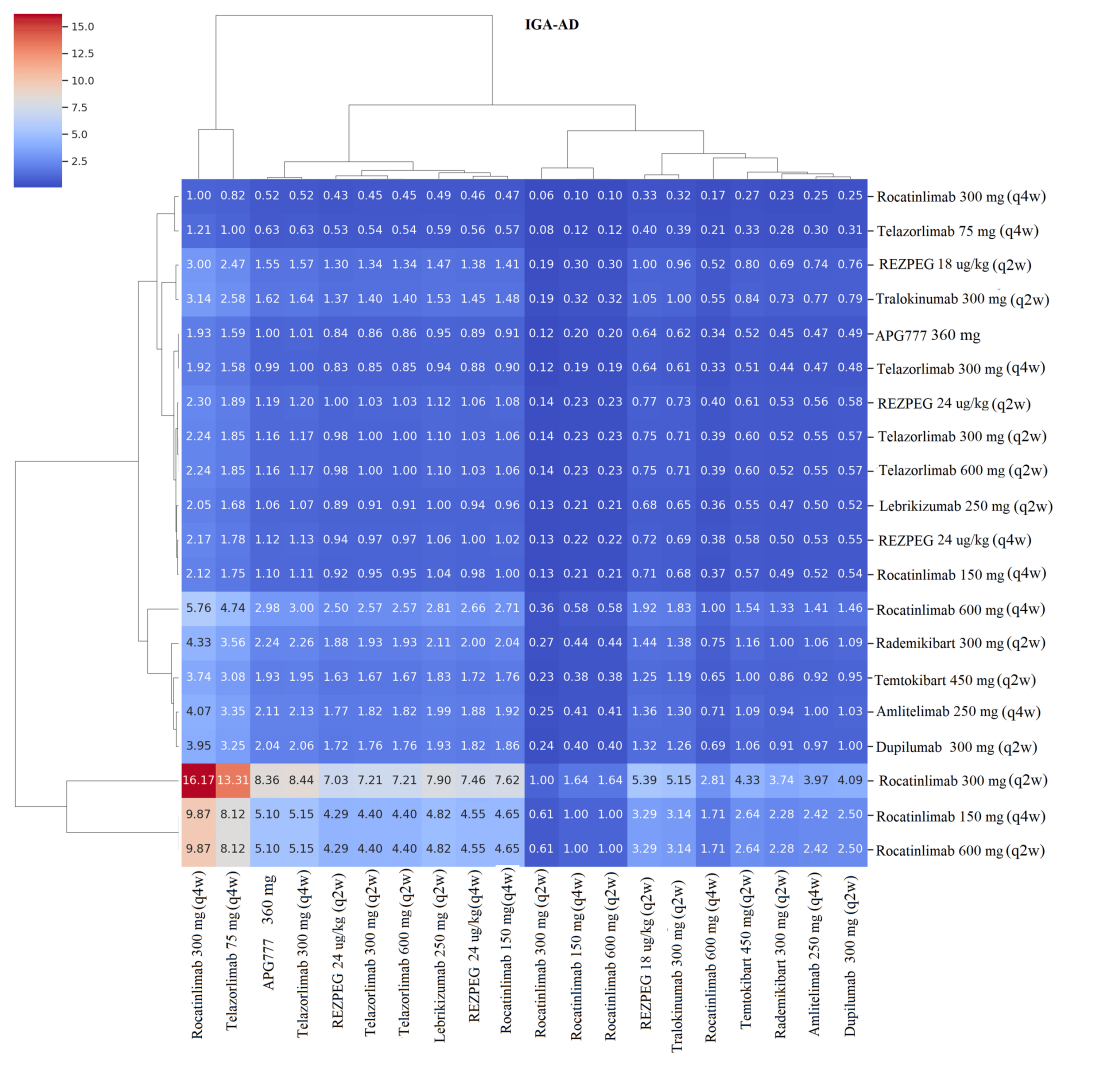
Clustered heatmap of pairwise risk ratios (RRs) for Investigator’s Global Assessment for Atopic Dermatitis (IGA-AD) response Heatmap presenting pairwise RRs for achieving IGA 0/1 response. Clustering highlights patterns of comparative efficacy among biologic and investigational therapies. Approved biologics were administered with loading doses (LDs) prior to maintenance therapy (dupilumab 600 mg LD → 300 mg (q2w); lebrikizumab 500 mg LD at Weeks 0 and 2 → 250 mg (q2w); tralokinumab 600 mg LD → 300 mg (q2w)), while investigational agents followed trial-specific induction regimens. Abbreviations: APG777, Zumilokibart; REZPEG; Rezpegaldesleukin. Advocate 1 (NCT04146363) [12]; Advocate 2 (NCT04178967) [12]; Phase 2b Study (NCT03443024) [11]; SOLO-1 (NCT02277743) [6]; SOLO-2 (NCT02277769) [6]; Phase 2b Study (NCT01859988) [5]; ECZTRA 1 (NCT03131648) [9]; ECZTRA 2 (NCT03160885) [9]; ECZTRA 6 (NCT03526861) [8]; Phase 2b Study (NCT05131477) [21]; OX40 (NCT03703102) [19, 20, 23]; IGNITE (NCT05398445) [20]; REZOLVE-AD (NCT06136741) [43]; APEX Phase 2 Part A (NCT06395948) [32]; CBP-201 (NCT05017480) [34]; Phase 2a Study (NCT04922021) [36]; Phase 2b Study (NCT03568162) [22]; q2w, every two weeks; q4w, every four weeks

**Figure 12:**
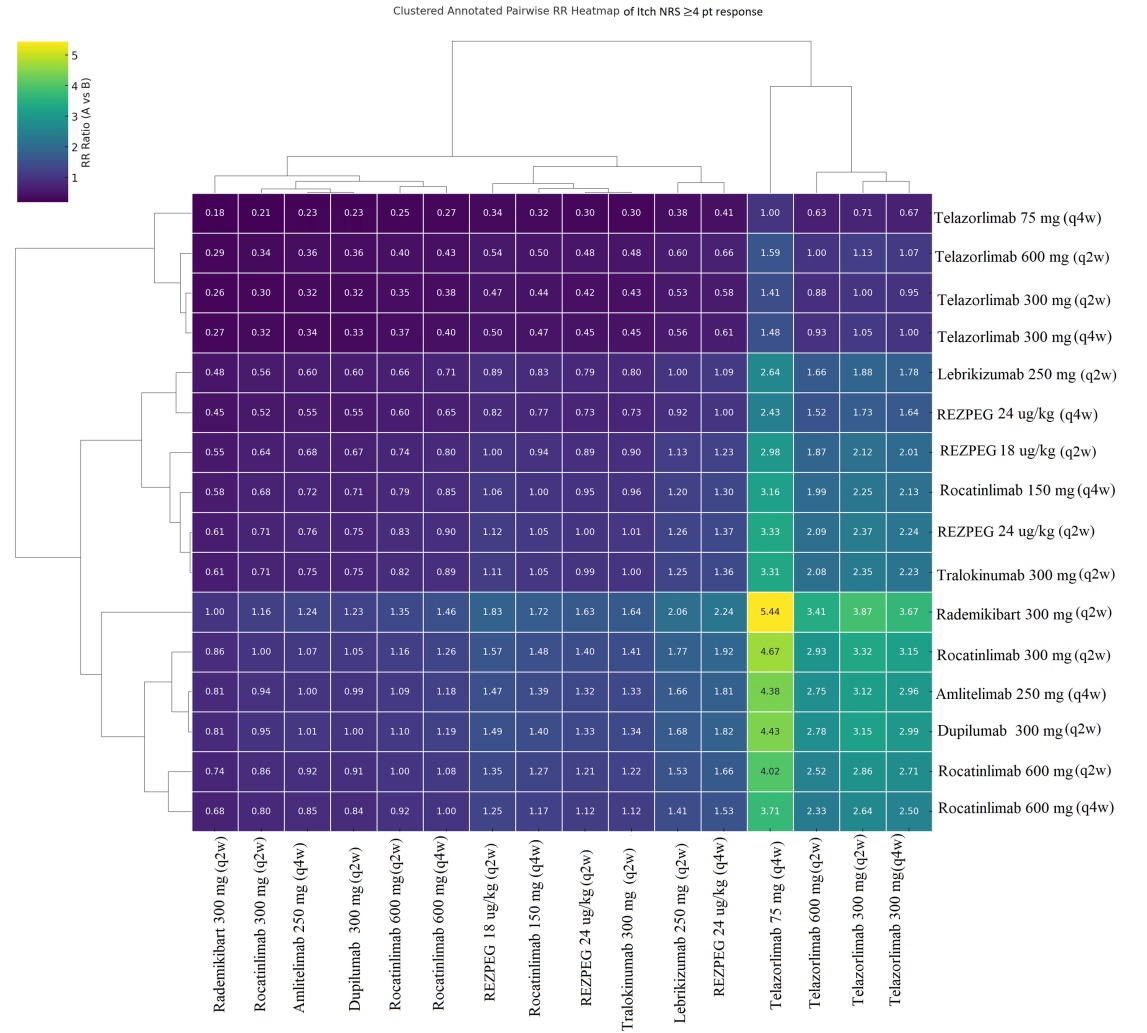
Clustered heatmap of pairwise risk ratios (RRs) for Numeric Rating Scale (NRS) ≥4-point improvement Clustered heatmap showing pairwise RRs for clinically meaningful itch reduction. Treatments are grouped by similarity in antipruritic efficacy. Comparisons reflect maintenance dosing, following loading doses for approved biologics and study-specific induction protocols for investigational agents. Abbreviation: REZPEG; Rezpegaldesleukin. Advocate 1 (NCT04146363) [12]; Advocate 2 (NCT04178967) [12]; Phase 2b Study (NCT03443024) [11]; SOLO-1 (NCT02277743) [6]; SOLO-2 (NCT02277769) [6]; Phase 2b Study (NCT01859988) [5]; ECZTRA 1 (NCT03131648) [9]; ECZTRA 2 (NCT03160885) [9]; ECZTRA 6 (NCT03526861) [8]; Phase 2b Study (NCT05131477) [21]; OX40 (NCT03703102) [19, 20, 23]; IGNITE (NCT05398445) [20]; REZOLVE-AD (NCT06136741) [43]; APEX Phase 2 Part A (NCT06395948) [32]; CBP-201 (NCT05017480) [34]; Phase 2a Study (NCT04922021) [36]; Phase 2b Study (NCT03568162) [22]; q2w, every two weeks; q4w, every four weeks

In all four clustered heatmap analyses (EASI-75, EASI-90, IGA-AD, and itch NRS≥4) (Figures 9-12), the ranking followed a consistent pattern in which dupilumab 300 mg (q2w) was again placed at the top with the largest risk ratio values compared to all comparators. Rocatinlimab 300 mg (q2w) was placed close to dupilumab, demonstrating consistent and uniform dominance over all other comparators with considerably reduced variability. The next cluster included rademikibart 300 mg (q2w), lebrikizumab 250 mg (q2w), and amlitelimab 250 mg (q4w), which showed moderate dominance with moderately large risk ratio values, though to a lesser extent than dupilumab and rocatinlimab. Rezpegaldesleukin treatment regimens and tralokinumab were placed in an intermediate zone with mixed dominance values. Telazorlimab treatment regimens were placed at the bottom in all four analyses, reflecting poor treatment dominance in all four analyses. Overall, all four analyses resulted in consistent data reinforcement in which dupilumab was placed at the top, followed closely by rocatinlimab.

Discussion

In this systematic review and BNMA of 17 RCTs evaluating 10 biologic monotherapies for moderate‑to‑severe AD, clear differences emerged in the efficacy of both established and investigational agents across clinically relevant endpoints. Across the evidence base, dupilumab (300 mg (q2w)), which blocks IL-4 and IL-13 signaling via IL-4Rα, remained the most consistently effective therapy, demonstrating predictable improvements in disease activity, skin clearance, and symptoms [5, 6, 13, 37, 40, 67, 68].

Rocatinlimab 300 mg (q2w), an anti‑OX40 antibody targeting activated CD4⁺ and CD8⁺ T cells, produced the strongest deep‑response signals, with the highest absolute EASI‑90, IGA 0/1, and itch‑response rates. However, its wide BNMA Crls indicate uncertainty driven by small sample sizes and early-phase data. Rademikibart, zumilokibart, and temtokibart also demonstrated favorable efficacy, with zumilokibart showing the highest EASI‑75 response among all agents. In contrast, rezpegaldesleukin exhibited dose- and frequency-dependent variability with reduced efficacy at shorter intervals, and telazorlimab showed consistently limited or unstable responses across endpoints [32-35].

These patterns were reinforced in the BNMA when both effect magnitude and precision were considered. Dupilumab retained the most stable and credible performance, outperforming both approved and investigational agents when accounting for Crl width and consistency. Although agents such as rocatinlimab and temtokibart occasionally produced numerically higher point estimates, their broad credible intervals substantially reduced confidence in their comparative superiority.

The SUCRA (P‑score) probability rankings further clarified comparative efficacy. Dupilumab ranked highest, reflecting its uniformly strong performance across all clinical outcomes. Rocatinlimab 300 mg (q2w) ranked closely behind, reinforcing its potential as an effective initial biologic pending phase 3 confirmation. Mid‑range agents, including rademikibart, lebrikizumab, temtokibart, and zumilokibart, showed moderate to high-SUCRA rankings. Rezpegaldesleukin and tralokinumab demonstrated intermediate rankings, whereas telazorlimab consistently ranked lowest, consistent with its comparatively limited clinical efficacy.

Pairwise risk‑ratio heatmaps added a complementary visual perspective, showing tight clustering of high‑performing biologics (dupilumab, rocatinlimab, rademikibart, and lebrikizumab) and weaker clustering of telazorlimab and rezpegaldesleukin. The consistent dominance of dupilumab and rocatinlimab across all four heatmaps highlights their superior and reproducible performance when compared head‑to‑head with other agents.

Safety remains a critical consideration when comparing biologics. Dupilumab, tralokinumab, and lebrikizumab have demonstrated favorable safety profiles in large phase 3 trials. All approved biologics for AD, particularly dupilumab, are associated with injection‑site reactions and ocular adverse effects historically described as conjunctivitis. More recently, the broader term “ocular surface disease” has been adopted to encompass the full spectrum of ocular manifestations, including conjunctivitis, blepharitis, keratitis, and dry eye [67-69].

Across investigational biologic therapies for moderate‑to‑severe AD, available data indicate generally favorable short‑term safety profiles with no major emerging signals; however, safety datasets remain substantially smaller and less mature than those for approved biologics. Rezpegaldesleukin has shown a reassuring profile in phase 1/2 studies, with treatment‑emergent adverse events largely mild or moderate, comparable to placebo, and without class‑related risks such as conjunctivitis or herpes viral reactivation [22, 26, 32-34, 69-73]. Although early findings are encouraging, longer‑term data and larger controlled trials are needed to fully characterize the risk-benefit profiles of these emerging agents.

A major strength of this NMA is its foundation in a comprehensive systematic review and feasibility assessment, providing a rigorous evidence base with limited risk of bias. Secondary analyses further supported the robustness of the findings. Bayesian baseline‑risk‑adjusted random‑effects models were used to account for between‑trial heterogeneity and variability in placebo responses [66, 74, 75].

To our knowledge, this is the first NMA to integrate phase 2 and phase 3 monotherapy data for lebrikizumab, dupilumab, rezpegaldesleukin, amlitelimab, tralokinumab, zumilokibart, telazorlimab, temtokibart, rademikibart, and rocatinlimab. It is also the first to include four‑week itch endpoints, which were not assessed in prior NMAs. The multinational scope of the included studies spanning Europe, North America, South America, Asia, and Oceania supports broad generalizability. Together, these strengths reinforce the internal validity of this NMA and its applicability across diverse clinical settings and geographic regions.

While methodologically rigorous, this analysis has several limitations. First, the absence of head‑to‑head studies among approved and investigational biologics restricts rankings to indirect, placebo‑anchored comparisons, although shared placebo arms supported a coherent evidence network. The network geometry enabled efficient Bayesian estimation, but the limited number of studies, particularly for investigational agents with only phase 2 data, constrained full exploration of random‑effects models and assessment of inconsistency. Second, heterogeneity across trials, including identified factors (ethnicity, skin color, geography, environment, AD severity) and unmeasured influences (exposome, genetic and immunologic variation), likely introduced residual confounding despite adjustment strategies, raising considerations for the transitivity assumption. Baseline differences in EASI, IGA, and itch NRS scores may also have affected treatment effects, although sensitivity and meta‑regression analyses yielded only marginal gains in precision and did not alter the overall treatment hierarchy.

Third, efficacy estimates in this NMA were largely confined to the 16‑week placebo‑controlled induction period, with the exception of rocatinlimab, which reported 24‑week data. Because most biologics continue to show incremental improvement beyond induction, often through 52 weeks or longer, the analysis cannot fully characterize differences in durability of response, deepening response, complete skin clearance (EASI-100), relapse patterns, or long-term safety, key considerations in a chronic, relapsing disease. Once placebo arms are discontinued and participants are re‑randomized or transitioned to active therapy, the network loses its common comparator, limiting the validity of indirect comparisons during maintenance. This structure implicitly assumes that all biologics, including mechanistically distinct agents, follow similar long‑term trajectories, an assumption that may not hold. For example, rezpegaldesleukin, the only Treg‑pathway agonist among the investigational agents evaluated, recently reported Phase 2b maintenance‑phase findings suggesting continued deepening of response with monthly or quarterly dosing and increases in complete clearance (EASI‑100) among patients who had already achieved EASI‑75 or vIGA‑AD 0/1 at entry. These results, which require confirmation in pivotal Phase 3 studies, illustrate that long-term trajectories may differ across mechanisms of action and underscore the need for head-to-head maintenance-phase trials [76].

Fourth, publication bias could not be formally assessed due to the small number of studies per outcome, and quality‑of‑life measures were excluded because instruments, timepoints, and reporting formats varied widely, preventing the construction of a consistent network. Future trials using harmonized quality of life endpoints are needed to enable more comprehensive comparisons. Finally, this BNMA evaluated monotherapy only, necessitating the exclusion of nemolizumab, which has not been studied as monotherapy in phase 3 trials and is indicated only with topical corticosteroids or calcineurin inhibitors. In clinical practice, biologics are frequently used alongside topical therapies, which may yield different response patterns. Accordingly, the relative rankings presented here should be viewed as tentative, and more definitive evidence will require trials that directly compare the combination with topical corticosteroids.

For dupilumab, robust efficacy at standard dosing, an extensive long-term safety record, demonstrated benefit across multiple atopic comorbidities (asthma, chronic rhinosinusitis with nasal polyps, eosinophilic esophagitis, and prurigo nodularis), and consistent performance across diverse populations reinforce its position as the most reliably validated therapy for sustained disease control. Multiple peer‑reviewed studies support the use of dupilumab as a first‑line option in patients with AD and comorbid asthma, which affects approximately 25% of patients and contributes meaningfully to overall morbidity. Collectively, these findings establish dupilumab as the benchmark biologic, providing durable skin clearance and itch reduction at its recommended dosing interval. Ultimately, selection of a biologic for moderate‑to‑severe AD must balance efficacy, comorbidity profiles, ocular surface disease risk, safety, and individual tolerability, considerations that shape long‑term adherence and clinical outcomes.

## Conclusions

This comprehensive BNMA provides the first unified comparison of all approved biologics alongside next‑generation investigational immunotherapies for moderate‑to‑severe AD. Across all efficacy endpoints, dupilumab 300 mg (q2w) consistently demonstrated the most reliable and reproducible improvements in skin clearance, global disease severity, and pruritus, supported by the strongest precision and longest‑standing evidence base. Rocatinlimab 300 mg (q2w) emerged as the leading investigational agent, achieving the highest deep‑response rates, although this assessment was based solely on short-term efficacy outcomes. SUCRA rankings and pairwise comparisons further reinforced a consistent efficacy hierarchy, with dupilumab and rocatinlimab occupying the top positions across outcomes. Together, these findings offer a comprehensive comparative framework to guide clinical decision‑making and therapeutic sequencing for biologic monotherapy in AD, while underscoring the need for future head‑to‑head trials, longer‑term safety evaluations, and mechanistic studies to define the optimal placement of next‑generation immunotherapies within the evolving treatment landscape.
